# The Left-Right Side-Specific Neuroendocrine Signaling from Injured Brain: An Organizational Principle

**DOI:** 10.1093/function/zqae013

**Published:** 2024-03-14

**Authors:** Hiroyuki Watanabe, Yaromir Kobikov, Olga Nosova, Daniil Sarkisyan, Vladimir Galatenko, Liliana Carvalho, Gisela H Maia, Nikolay Lukoyanov, Igor Lavrov, Michael H Ossipov, Mathias Hallberg, Jens Schouenborg, Mengliang Zhang, Georgy Bakalkin

**Affiliations:** Department of Pharmaceutical Biosciences, Uppsala University, Uppsala, SE-751 24, Sweden; Department of Molecular Medicine, University of Southern Denmark, Odense, DK-5230, Denmark; Volunteer Associate at Department of Pharmaceutical Biosciences, Uppsala University, Uppsala, SE-751 24, Sweden; Department of Pharmaceutical Biosciences, Uppsala University, Uppsala, SE-751 24, Sweden; Department of Pharmaceutical Biosciences, Uppsala University, Uppsala, SE-751 24, Sweden; Department of Immunology, Genetics and Pathology and Science for Life Laboratory, Uppsala University, Uppsala, SE-751 08, Sweden; Evotec International GmbH, Göttingen 37079, Germany; Departamento de Biomedicina da Faculdade de Medicina da Universidade do Porto, Porto 4200-319, Portugal; Centro de Investigação em Saúde Translacional e Biotecnologia Médica (TBIO)/Rede de Investigação em Saúde (RISE-Health), Escola Superior de Saúde, Instituto Politécnico do Porto, Porto 4200-072, Portugal; Medibrain, Vila do Conde 4480-807, Portugal; Brain Research Institute, Porto 4450-208, Portugal; Departamento de Biomedicina da Faculdade de Medicina da Universidade do Porto, Porto 4200-319, Portugal; Brain Research Institute, Porto 4450-208, Portugal; i3S—Instituto de Investigação e Inovação em Saúde, Universidade do Porto, Porto 4200-135, Portugal; Department of Neurology, Mayo Clinic, Rochester, MN 55905, USA; Department of Pharmacology, University of Arizona College of Medicine, Tucson, AZ 85724-5050, USA; Department of Pharmaceutical Biosciences, Uppsala University, Uppsala, SE-751 24, Sweden; Neuronano Research Center, Department of Experimental Medical Science, Lund University, Lund 223 63, Sweden; Department of Molecular Medicine, University of Southern Denmark, Odense, DK-5230, Denmark; Neuronano Research Center, Department of Experimental Medical Science, Lund University, Lund 223 63, Sweden; Department of Pharmaceutical Biosciences, Uppsala University, Uppsala, SE-751 24, Sweden

**Keywords:** humoral signaling, neuroendocrine system, gene co-expression networks, left-right patterns, brain injury, contralateral effects, motor deficits, postural asymmetry

## Abstract

A neurological dogma is that the contralateral effects of brain injury are set through crossed descending neural tracts. We have recently identified a novel topographic neuroendocrine system (T-NES) that operates via a humoral pathway and mediates the left-right side-specific effects of unilateral brain lesions. In rats with completely transected thoracic spinal cords, unilateral injury to the sensorimotor cortex produced contralateral hindlimb flexion, a proxy for neurological deficit. Here, we investigated in acute experiments whether T-NES consists of left and right counterparts and whether they differ in neural and molecular mechanisms. We demonstrated that left- and right-sided hormonal signaling is differentially blocked by the δ-, κ- and µ-opioid antagonists. Left and right neurohormonal signaling differed in targeting the afferent spinal mechanisms. Bilateral deafferentation of the lumbar spinal cord abolished the hormone-mediated effects of the left-brain injury but not the right-sided lesion. The sympathetic nervous system was ruled out as a brain-to-spinal cord-signaling pathway since hindlimb responses were induced in rats with cervical spinal cord transections that were rostral to the preganglionic sympathetic neurons. Analysis of gene–gene co-expression patterns identified the left- and right-side-specific gene co-expression networks that were coordinated via the humoral pathway across the hypothalamus and lumbar spinal cord. The coordination was ipsilateral and disrupted by brain injury. These findings suggest that T-NES is bipartite and that its left and right counterparts contribute to contralateral neurological deficits through distinct neural mechanisms, and may enable ipsilateral regulation of molecular and neural processes across distant neural areas along the neuraxis.

## Graphical Abstract

**Graphical Abstract:**
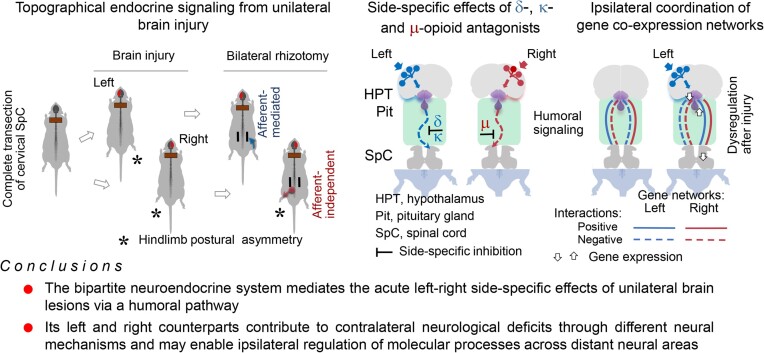

## Introduction

The central neurology dogma known as *cross association* states that each cerebral hemisphere is functionally connected to the contralateral side of the body through the decussating neural tracts.^1–6^ Traumatic brain injury and stroke in patients, and brain lesions in animal experiments cause postural and sensorimotor deficits that are generally contralateral and include asymmetric posture and reflexes.^7–11^ After a unilateral injury to the hindlimb sensorimotor cortex, animals exhibit hindlimb postural asymmetry (HL-PA) with flexion of the limb contralateral to the lesion (ie, contralesional), and asymmetry of reflexes with greater activity on the contra- vs. ipsilesional side.^11–14^ A cause of the contralateral effects of brain lesions has been considered as solely neuroanatomical—based on the decussation of the descending neural pathways.^6^,^15^,^16^

Emerging evidence indicates that, in addition to neural mechanisms, the contralateral effects of a unilateral brain injury (UBI) are mediated through the humoral pathway by neurohormones that produce either the left- or right-side-specific effects.^6^,^12–14^ This humoral signaling was identified in animals whose descending neural tracts were disabled by complete transection of the spinal cord that was then followed by a brain lesion. Strikingly, rats with transected thoracic spinal cords and unilateral injuries of the hindlimb sensorimotor cortex developed contralateral hindlimb flexion, asymmetry in hindlimb withdrawal reflexes, and asymmetric changes in gene expression patterns in the lumbar spinal cord. Left-side brain injury resulted in right hindlimb flexion, while injury of the right hemisphere induced the left hindlimb flexion. Hypophysectomy abolished these effects, whereas serum from animals with UBI injected into rats with intact brains induced HL-PA in those animals. Arg-vasopressin and β-endorphin were identified as molecules that mediate the effects of the left-sided brain injury. They are produced in the hypothalamic–pituitary system and evoke HL-PA with right hindlimb flexion in animals with intact brain.^12^ Thus, the left-right side-specific neuroendocrine signals may bypass descending neural tracts and convey information on the side of brain injury. These neurohormones, released from the hypothalamus or pituitary gland, could be a part of a general mechanism that spans the nervous system, or even the entire body, and thus enables differential neuroendocrine control of the left and right body sides. From a clinical standpoint, this phenomenon may contribute to asymmetric neurological deficits secondary to stroke and traumatic brain injury and may be pharmacologically targeted by neurohormonal antagonists. How this topographic left-right side-specific neuroendocrine system (T-NES) is organized and functions is still an enigma. Three stages may be envisaged: the encoding of signals from the left and right hemispheres into side-specific neurohormones in the hypothalamus and pituitary gland, the release of these neurohormones into the blood, and the decoding of these hormonal messages into left-right-sided responses in the spinal cord and peripheral nervous system.^6^,^12^ Differential encoding of top-down signaling from two anatomically symmetric hemispheres requires bipartite, lateralized, and hemisphere (side) specific organization of the T-NES.

We reasoned that the T-NES consists of two counterparts that differentially process and convey the left and right side-specific messages, that their activities are balanced in intact rats, and that this balance may be perturbed by a UBI. These two parts may be mirror-symmetric in their structure, (eg, in cell type composition and connectivity), or they may differ in their internal architecture and exploit different endocrine, neural, and molecular mechanisms to produce symmetric physiological outcomes.

In this study, we addressed these hypotheses with an aim to characterize the T-NES counterparts and to reveal their lateralized features. The HL-PA, a proxy for neurological deficit with binary left- or right-sided outcomes including directional asymmetry in posture and motor functions,^11–14^,^17^ was used to characterize and compare the left and right T-NES counterparts in acute experiments. To analyze the T-NES, neural pathways between the brain and lumbar spinal cord were disabled by complete spinal cord transection at the cervical level. These transections were rostral to the thoracic preganglionic sympathetic neurons and allowed us to examine the paravertebral sympathetic chain of ganglia as the signaling pathway from the brain to the lumbar spinal cord. The left or right hindlimb sensorimotor cortex was injured to evoke signaling through the left or right T-NES counterparts in order to separately analyze their features. The cortex was injured by ablation in order to restrict the injured area to the hindlimb sensorimotor cortex and to examine specific changes in lumbar spinal circuits and hindlimb motor functions. In this biologically relevant acute injury model pathological factors that may interfere with the T-NES functions (eg, neuroinflammation, widespread damage to neurons, axons, and blood vessels) that are produced by traumatic brain injury and stroke^18^,^19^ could be largely excluded.

Analysis of signaling from the sensorimotor cortex injured on the left- or right-side demonstrated that the T-NES is binary and functionally asymmetric; the left- or right-side T-NES counterparts differently target the contralateral afferent systems controlling hindlimb functions. Experiments with opioid receptor antagonists confirmed that the T-NES is bipartite and that the messages from the left and the right hemispheres are differentially controlled through the δ-, κ-, and µ-receptors. Analysis of gene expression suggested that UBI affects the hypothalamus and pituitary gland, that the side-specific molecular processes are coordinated between the hypothalamus and the lumbar spinal cord by the T-NES, and that this coordination is ipsilateral and impaired by UBI.

## Materials and Methods

### Animals

Adult male Sprague Dawley rats (Taconic, Denmark) weighing 190–410 g were used in the study. The animals received food and water ad libitum and were kept in a 12-h day–night cycle (light on from 10:00 pm to 10:00 am) at a constant environmental temperature of 21°C (humidity: 65%) and randomly assigned to their respective experimental groups. Approval for animal experiments was obtained from the Malmö/Lund Ethical Committee on Animal Experiments (No. M7–16). Experiments were performed from 9:00 am to 8:00 pm. After the experiments were completed, the animals were given a lethal dose of pentobarbital.

### Spinal Cord Transection

The animals were anesthetized with sodium pentobarbital anesthesia (intraperitoneal, I.P.; 40 mg/kg body weight, as an initial dose and then 6 mg/kg every hour). Core temperature of the animals was controlled using a feedback-regulated heating system.

The experimental design included rats with UBI, which was preceded by a complete spinal cord transection. Anaesthetized animals were mounted onto the stereotaxic frame, and the skin of the back was incised along the midline at the level of the superior thoracic vertebrae. After the back muscles were retracted to the sides, a laminectomy was performed at the C6 and C7 vertebrae. A 3–4-mm spinal cord segment between the two vertebrae was dissected and removed.^12^ The completeness of the transection was confirmed by (i) inspecting the cord during the operation to ensure that no spared fibers bridged the transection site and that the rostral and caudal stumps of the spinal cord were completely retracted; and (ii) examining the spinal cord in all animals after termination of the experiment.

### Brain Surgery

The head of the rats mounted onto the stereotaxic frame was fixed in a position in which the bregma and lambda were located at the same horizontal level. After local injection of lidocaine (Xylocaine, 3.5 mg/mL) with adrenaline (2.2 µg/mL), the scalp was cut open, and a piece of the parietal bone located 0.5–4.0 mm posterior to the bregma and 1.8–3.8 mm lateral to the midline^20^ was removed. The part of the cerebral cortex located below the opening that includes the hind-limb representation area of the sensorimotor cortex was aspirated with a glass pipette (tip diameter 0.5 mm) connected to an electrical suction machine (Craft Duo-Vec Suction Unit, Rocket Medical Plc, UK). Care was taken to avoid damaging the white matter below the cortex. After the ablation, bleeding was stopped with a piece of Spongostone, and the bone opening was covered with a piece of TissuDura (Baxter, Germany). For sham operations, animals underwent the same anesthesia and surgical procedures, but the cortex was not ablated.

After completion of all surgical procedures, the wound was closed with 3-0 suture (AgnTho’s, Sweden), and the rat was kept under an infrared radiation lamp to maintain appropriate body temperature during monitoring of postural asymmetry and during stretching force analysis.

### Dorsal Rhizotomy

A bilateral dorsal rhizotomy was performed in rats with complete transection of the cervical spinal cords 3 h after the UBI. After laminectomy from the T11 to L3 vertebral level, the dura was opened, and the dorsal roots were cut bilaterally from the L1 to S2 spinal levels with a pair of fine scissors as close to their exit as possible from the spinal column so that the spinal cord was not damaged. After each cut, the dorsal rootlets were flipped to make sure that the rhizotomy was complete. This procedure prevents hindlimb afferent input to the spinal cord as demonstrated elsewhere.^21–24^ The HL-PA and stretching resistance were analyzed before UBI, 3 h after UBI before rhizotomy, and 0.5 h after rhizotomy.

### Histological Analysis of Brain Injury

Localization and size of cortical lesions were analyzed in rats with left side (*n* = 5) and right side (*n* = 5) UBI 3–5.5 h after the injury. After perfusion with 4% paraformaldehyde, the brain was removed and postfixed in the same fixative overnight. Then the brain was soaked in phosphate-buffered saline with 30% sucrose for 48 h, dissected into blocks, which were then sliced into 50 µm sections with a freezing microtome. Every fourth section was stained with toluidine (Nissl stain), and all the stained sections across the lesion site were photographed, and the rostrocaudal respective mediolateral extension as well as lesion volume were calculated.

### Analysis of HL-PA by the Hands-On and Hands-Off Methods

The HL-PA value and the side of the flexed limb were assessed as described elsewhere.^11^,^12^,^14^ Briefly, the measurements were performed under pentobarbital anesthesia (40 mg/kg, i.p.). The level of anesthesia was characterized by a barely perceptible corneal reflex and a lack of overall muscle tone. The anesthetized rat was placed in the prone position on the 1-mm grid paper.

In the hands-on analysis, the hip and knee joints were straightened by gently pulling the hindlimbs backwards for 1 cm to reach the same level. Then, the hindlimbs were set free, and the magnitude of postural asymmetry (MPA) was measured in millimeters as the length of the projection of the line connecting symmetric hindlimb distal points (digits 2–4) on the longitudinal axis of the rat. The procedure was repeated 6 times in immediate succession.

In the hands-off method, silk threads were glued to the nails of the middle 3 toes of each hindlimb, and their other ends were tied to 1 of two hooks attached to the movable platform that was operated by a micromanipulator constructed in the laboratory.^12^ To reduce potential friction between the hindlimbs and the surface with changes in their position during stretching and after releasing them, the bench under the rat was covered with plastic sheet and the movable platform was raised up to form a 10° angle between the threads and the bench surface. The limbs were adjusted to lie symmetrically, and stretching was performed over a distance of 1.5 cm at a rate of 2 cm/s. The threads then were relaxed, the limbs were released, and the resulting HL-PA was photographed. The procedure was repeated 6 times in succession, and the HL-PA values for a given rat were used in statistical analyses.

The limb that projected over a shorter distance from the trunk was considered to be flexed. The HL-PA was measured in mm with negative and positive HL-PA values that were assigned to rats with the left and right hindlimb flexion, respectively. This measure, the postural asymmetry size (PAS), shows the HL-PA value and flexion side. The PAS does not show the proportion of the animals with asymmetry in each group, whether all or a small fraction of animals display the asymmetry; and cannot be used for analysis of rat groups with the similar number of left or right flexion. In the latter case, the HL-PA value would be about zero. Therefore, the HL-PA was also assessed by the MPA that shows absolute flexion size, and the probability of postural asymmetry (*P*_A_) that shows the proportion of animals exhibiting HL-PA at the imposed threshold (>1 mm). The MPA and *P*_A_ do not show a flexion side. These 3 measures are obviously dependent; however, they are not redundant and for this reason, all are required for characterization of the HL-PA data and presentation.

### Analysis of Hindlimb Resistance to Stretch

Stretching force was analyzed under pentobarbital anesthesia within 3–5 h after UBI using the micromanipulator-controlled force meter device constructed in the laboratory.^11^ Two Mark-10 digital force gauges (model M5-05, Mark-10 Corporation, USA) with a force resolution of 50 mg were fixed on a movable platform operated by a micromanipulator. Three 3-0 silk threads were glued to the nails of the middle 3 toes of each hindlimb, and their other ends were hooked to 1 of 2 force gauges. The flexed leg of the rat in the prone position was manually stretched to the level of the extended leg; this position was taken as 0 mm point. Then both hindlimbs were stretched caudally, moving the platform by micromanipulator at a constant rate of 5 mm/s for 10 mm. No, or very little, trunk movement was observed with stretching for the first 10 mm; therefore, the data recorded for this distance were included in statistical analysis. The forces (in grams) detected by each of the 2 gauges were simultaneously recorded (100 Hz frequency) during stretching. Five successive ramp-hold-return stretches were performed as technical replicates. Because the entire hindlimb was stretched, the measured resistance was characteristic of the passive musculo-articular resistance integrated for hindlimb joints and muscles.^25–27^ The resistance analyzed could have both neurogenic and mechanical components, but their respective contributions were not distinguished in the experimental design. The resistance was measured as the amount of mechanical work *W*_L_ and *W*_R_ to stretch the left and right hindlimbs, where *W* was stretching force integrated over the stretching distance interval from 0 to 10 mm.

### Drug Treatment Design

Nor-binaltorphimine (BNI; 6 mg/kg) and β-funaltrexamine (FNA; 3 mg/kg) were administered subcutaneously 1 d before the spinal cord transections and brain surgeries. Naloxone (10 mg/kg), naltrindole (NTI; 5 mg/kg), and saline were administered intraperitoneally 3–4 h after UBI into rats with the MPA > 1.5 mm, and their effects on HL-PA were measured 1 h later.

Doses and timeline for naloxone,^28^ NTI,^29–31^ BNI,^31–33^ and FNA^30^ required to block the respective receptors were robustly established in previous studies. The dose for naloxone was chosen to block all 3 subtypes of opioid receptors. BNI and FNA exert long-lasting antagonistic effects that persist for at least 1 mo and are receptor selective from 24 h after administration. The antagonists were purchased from Tocris (Minneapolis, MN). All test compounds were dissolved in saline.

### Analysis of Gene Expression

Gene expression was analyzed in the pituitary gland, and in the left and right halves of the hypothalamus and of the lumbar spinal cord. These tissues were collected 3 h after left UBI (*n* = 12) or left sham surgery (*n* = 11) that was performed in rats with complete transection of the spinal cord. The tissue samples were snap frozen and stored at −80°C until assay.

#### Quantitative Real-Time PCR

Total RNA was purified by using the RNeasy Lipid Tissue Mini Kit (Qiagen, Valencia, CA, USA). RNA concentrations were measured with Nanodrop (Nanodrop Technologies, Wilmington, DE, USA). RNA (500 ng) was reverse-transcribed to cDNA with the cDNA iScript Kit (Bio-Rad Laboratories, CA, USA) according to manufacturer’s protocol. cDNA samples were aliquoted and stored at –20°C. cDNAs were mixed with PrimePCR™ Probe assay and iTaq Universal Probes supermix (Bio-Rad) for qPCR with a CFX384 Touch™ Real-Time PCR Detection System (Bio-Rad Laboratories, CA, USA) according to manufacturer’s instructions. TagMan assay was performed in 384-well format with TagMan probes that are listed in Figure 4—figure supplements S1–S4, S7.

All procedures were conducted strictly in accordance with the established guidelines for the qRCR based analysis of gene expression, consistent with the minimum information for publication of quantitative real-time PCR experiments guidelines.^34^,^35^ The raw qPCR data were obtained by the CFX Maestro™ Software for CFX384 Touch™ Real-Time PCR Detection System (Bio-Rad Laboratories, CA, USA). The mRNA levels of genes of interest were normalized to the geometric mean of expression levels of 2 reference genes, *Actb* and *Gapdh*. GeNorm software was used to analyze the gene expression stability (*M-*value) of the 10 candidate reference genes (*Actb, B2m, Gapdh, Gusb, Hprt, Pgk, Ppia, Rplpo13a, Tbp*, and *Tfrc*). The calculation of the *M*-value was based on the pairwise variation between 2 reference genes. If the *M-*value was less than 1.5, it could be considered as a suitable reference gene. The smaller the *M-*value, the higher the stability of gene expression levels (https://genorm.cmgg.be/ and^36^). The expression stability of candidate reference genes was computed for all sets of samples and identified *Actb* and *Gapdh* as the most stably expressed genes. For all 3 regions analyzed, the gene expression stability (*M-*values) did not exceed 0.5. The optimal number of reference genes was determined by calculating pairwise variation (*V-*value) by geNorm program. The *V-*value for *Actb* and *Gapdh*, the top reference genes, was 0.12 that did not exceed the 0.15 threshold demonstrating that analysis of these 2 genes is sufficient for normalization.

#### Hormonal, Neurohormonal, and Neuroplasticity-Related Genes

Genes analyzed in the hypothalamus included the hypothalamic neurohormone and neuropeptide genes (Figure 4—figure supplement S1; genes of corticotropin releasing hormone *Crh*, growth hormone releasing hormone *Ghrh*, gonadotropin releasing hormone 1*Gnrh1*, neurotensin *Nts*, somatostatin *Sst* and thyrotropin releasing hormone *Trh*); genes of the endogenous opioid system (Figure 4—figure supplement S2; genes of δ-opioid receptor *Oprd1*, κ-opioid receptors *Oprk1*, µ-opioid receptors *Oprm1*, prodynorphin *Pdyn*, proenkephalin *Penk*, and proopiomelanocortin *Pomc*) and the oxytocin-vasopressin system (Figure 4—figure supplement S3; genes of arginine vasopressin *Avp* and oxytocin *Oxt*); and neuroplasticity-related genes (Figure 4—figure supplement S4). The *Avpr1a, Avpr1b*, and *Avpr2* genes listed in Figure 4—figure supplement S3 were expressed at low levels and were excluded from further analysis.

Genes selected as neuroplasticity-related were identified as such in several major studies. The selection of each gene from this set is justified by referring to these studies (see references below) and is not biased. To note, there is no established view on how to categorize genes as neuroplasticity-related, and there are no lists of neuroplasticity-related genes consistent among studies. Thus, such a selection is arbitrary, and a set of selected genes could not be comprehensive. The selected neuroplasticity-related genes were *Arc*, activity-regulated cytoskeletal gene implicated in numerous plasticity paradigms; *Bdnf*, brain-derived neurotrophic factor regulating synaptogenesis; *cFos*, a neuronal activity-dependent transcription factor; *Dlg4* gene encoding PSD95 involved in AMPA receptor-mediated synaptic plasticity and post NMDA receptor activation events; *Egr1* regulating transcription of growth factors, DNA damage, and ischemia genes; *Gap-43* coding for growth-associated protein Gap-43 that regulates axonal growth and neural network formation; *GluR1* and *Grin2b* coding for the glutamate ionotropic receptor AMPA Type Subunit 1 and NMDA receptor subunit, respectively, both involved in glutamate signaling and synaptic plasticity; *Grin2a* subunit of the glutamate receptors that regulates formation of neural circuits and their plasticity; *Homer-1* giving rise to homer scaffold protein 1, a component of glutamate signaling involved in nociceptive plasticity; *Pcsk6* gene encoding proprotein convertase subtilisin/kexin type 6 involved in post-translational modification; *Nfkbia* (I-Kappa-B-Alpha) that inhibits NF-kappa-B/REL complexes regulating activity-dependent inhibitory and excitatory neuronal function; *Syt4* (Synaptotagmin 4) playing a role in dendrite formation and synaptic growth and plasticity; and *Tgfb1* that gives rise to transforming growth factor β1 regulating inflammation, expression of neuropeptides, and glutamate neurotoxicity^37–52^ (Figure 4—figure supplement S4).

Genes coding for pituitary hormones (Figure 4—figure supplement S7; gene of alpha polypeptide of glycoprotein hormones *Cga*, follicle stimulating hormone, subunit beta *Fshb*, glycoprotein hormones, alpha subunit *Gh1*, luteinizing hormone *Lhb*, prolactin *Prl*, and thyroid stimulating hormone, beta subunit *Tshb*) and neuropeptides and their receptors (Figure 4—figure supplements S2, S3) were analyzed in the pituitary gland.

In the spinal cord, neuroplasticity-related genes (Figure 4—figure supplement S4) along with the neuropeptide and their receptor genes (Figure 4—figure supplements S2 and S3) were analyzed besides the *Avp, Avpr1b, Avpr2, Oxt*, and *Pomc* genes that were expressed at low levels.

### Statistical Analysis

Experimental data were processed and statistically analyzed after completion of the experiments by the statisticians who were not involved in their execution. No intermediate assessment was performed to avoid any bias in data acquisition. Experimenters were not blind because the signs of the asymmetry were evident after brain injury, and the UBI-animals with asymmetry exceeding 1.5 mm were selected for analysis of the antagonists. The asymmetry data were obtained by unbiased hand-off method and unbiased registration of stretching force.

#### Processing of Physiological Data

##### Bayesian Framework

Predictors and outcomes scaled and centered before we fitted Bayesian regression models via full Bayesian framework by calling *Stan 2.21.7* (Stan Development Team 2022). RStan: the R interface to Stan. R package version 2.21.7 (https://mc-stan.org/) from *R* 4.1.3 (R Core Team 2022). R: A language and environment for statistical computing. R Foundation for Statistical Computing, Vienna, Austria. URL (https://www.R-project.org/) using the *brms* 2.18^53^ interface. To reduce the influence of outliers, models used Student’s *t* response distribution with identity link function unless explicitly stated otherwise. Models had no intercepts with indexing approach to predictors.^54^ Default priors were provided by the *brms* according to Stan recommendations.^55^ Intercepts, residual SD, and group-level SD were determined\from the weakly informative prior Student_t (3, 0, 10). The additional parameter *ν* of Student’s distribution representing the degrees of freedom was obtained from the wide gamma prior gamma(2, 0.1). Group-level effects were determined from the very weak informative prior normal (0, 10). Four MCMC chains of 40 000 iterations were simulated for each model, with a warm-up of 20 000 runs to ensure that effective sample size for each estimated parameter exceeded 10 000^56^ producing stable estimates of 95% highest posterior density credible intervals (HPD). MCMC diagnostics were performed according to the Stan manual. *P*-values, adjusted using the multivariate *t* distribution with the same covariance structure as the estimates, were produced by frequentist summary in *emmeans* 1.8.4-1^57^ together with the medians of the posterior distribution and 95% HPD. The asymmetry and contrast between groups were defined as significant if the corresponding 95% HPD did not include zero and the adjusted *P*-value was ≤.05.

##### Postural Asymmetry

The MPA was inferred via Bayesian framework using Gaussian response distribution. The probability of HL-PA (*P*_A_) was inferred via Bayesian framework with Bernoulli response distribution and logit link function.

##### Stretching Force

The amount of mechanical work *W*_L_ and *W*_R_ to stretch the left and right hindlimbs, respectively, was computed by integrating the smoothed stretching force measurements over stretching distance from 0 to 10 mm using loess smoothing computed by *loess* function from R package *stats* with parameters span = 0.4 and family = “symmetric.” Asymmetry was assessed both as the left/right asymmetry index AI_L/R_ = log_2_ (*W*_L/_*W*_R_), the contra- and ipsilesional asymmetry index AI_C/I_ = log_2_ (*W*_C/_*W*_I_), and as the difference in work between left and right hindlimbs *W*_L-R_ = (*W*_L_ – *W*_R_) and between contra- and ipsilesional hindlimbs *W*_C-I_ = (*W*_C_ − *W*_I_). The AI and the difference in *W* were inferred via Bayesian framework by fitting linear multilevel models that included *operation type* (left UBI vs. right UBI vs. sham) as the factor of interest.

#### Molecular Analysis

##### Expression Levels

The Lilliefors and Levene’s tests revealed deviations from normality and differences in the variances between the rat groups, respectively, for the expression levels and the asymmetry index of several genes in each rat group. The mRNA levels were compared separately for the pituitary gland and left and right halves of the hypothalamus between UBI or sham surgery groups using Mann–Whitney test followed by Bonferroni correction for a number of tests (*n* = 17 and 56, respectively). FC was computed as a ratio of median expression levels in the UBI to sham groups.

The asymmetry index (AI_L/R _= median[log_2_L/R)], where L and R were gene expression levels in the left and right halves of hypothalamus or spinal cord, respectively), was computed for each gene in each area (Figures 4 and 5), and compared between UBI and sham surgery groups using Mann–Whitney test followed by a Bonferroni correction for multiple tests (*n* = 28 and 20 for the hypothalamus and spinal cord, respectively). Because no significant differences between UBI and sham surgery groups were revealed, the groups were combined, and the pooled data were used for analysis of lateralization of gene expression. One-sample version of non-parametric Wilcoxon signed-rank test was applied to compare the AI_L/R_ with zero, followed by Bonferroni multiple testing correction (28 genes for hypothalamus). Data for the spinal cord were acquired and analyzed in our previous study.^12^

Linear model fitting and analysis were performed in R using *lm, summary.lm*, and *confint* commands. Differences were considered to be significant if the *P*-value corrected for multiple testing (*P*_adjusted_) did not exceed .05.

##### Gene–Gene Co-Expression Patterns

In the hypothalamus and spinal cord separately, genes were categorized into 2 groups that were defined as the left (LdN; AI_L/R_ > 0) and right (RdN; AI_L/R_ < 0) dominant gene co-expression networks (Figure 4—figure supplement S9; Figure 5—figure supplement S1). Three categorization variants were used in the following correlation analysis. Genes were assigned into 2 groups: (i) by their median AI_L/R_ in the combined UBI and sham surgery group (variant 1); and (ii) by their median AI_L/R_ in the sham surgery group only (variant 2); and (iii) by their mean AI_L/R_ in the combined UBI and sham surgery group (variant 3). Three variants were separately applied for analysis of correlation patterns in each the hypothalamus and spinal cord, and between these areas (*source data*: the EXCEL source data files “Table III-S6 23 05 10.xlsx”). In the hypothalamus, all genes showed stable patterns between the LdN and RdN in 3 categorization variants besides *Mor* that wobbled. In the spinal cord, 5 LdN genes and 8 RdN genes showed stable patterns across the 3 variants, while 7 genes wobbled between the sides. The significant contrast: the *P*-value was ≤ .05 for (i) all 3 variants after correction, or for (ii) any 2 of them, while for the third variant it was <.05 and <.10 before and after the correction, respectively.

The correlation structure (gene–gene co-expression pattern) for each area, side, between the sides, and across the areas was examined using the Spearman’s rank correlation coefficient calculated for all gene pairs. The pattern of interactions between genes was characterized by the coordination strength (magnitude of correlations or the absolute value of the correlation coefficient averaged across pairwise correlations) and the proportion of positive correlations.

Robust and unbiased *P-*values were determined in the absence of distributional assumptions by permutation testing. A permutation procedure was employed to characterize the distribution of each statistical test under the null hypothesis of non-replication and non-preservation. Permutation test^58^ with *R* = 10^6^ bootstrap replicates implemented in the R/boot package was used to analyze the data. To generate null distribution, we permuted the data across (i) *rat identification numbers (IDs)*, (ii) *Treatment (UBI and sham surgery)*, (iii) *Module* (left and right) within each individual rat; and (iv) *CNS area* (hypothalamus and spinal cord) within each module.

The R/boot.pval package was used to compute the *P*-value.^59^  *P*-values were adjusted using Benjamini–Hochberg family wise multiple test correction that was applied separately for all tasks (source data: the EXCEL source data files “Table III-S6 23 05 10.xlsx”). Three sets were designed to compare the coordination strength, and the other 3 sets to compare the proportion of positive correlations. Two sets for both the hypothalamus and spinal cord were constructed for analysis of intra-area correlations in the strength and the proportion, and 2 sets for analysis of the inter-area correlations between the hypothalamus and spinal cord in the strength and the proportion. Each set included comparisons between UBI and sham surgery groups.

*Executed tasks* (Figures 4– 6; Figure 4—figure supplements S10 and S11; Figure 5—figure supplement S2; Figure 6—figure supplement S1; (*source data*: the EXCEL source data files “Table III-S6 23 05 10.xlsx”; raw_groups.xlsx).

*Task 1*. Pairwise gene–gene correlations within each the left (lmLdN-lmLdN vs. lmRdN-lmRdN vs. lmLdN-lmRdN correlations) and right (rmLdN-rmLdN vs. rmRdN-rmRdN vs. rmLdN-rmRdN correlations) modules were compared in each area separately to assess differences in the intra-modular coordination between the gene networks (Figures 4 and 5).

*Task 2*. Pairwise gene–gene intra-modular correlations internal for each the left and right gene networks were compared between the left and right modules lm and rm, respectively (lmLdN-lmLdN vs. rmLdN-rmLdN; lmRdN-lmRdN vs. rmRdN-rmRdN correlations) in each area separately to assess differences in the intra-modular coordination for each gene network between left and right modules (Figures 4 and 5).

*Task 3*. Pairwise gene–gene inter-modular correlations internal for each the left and right gene networks, were compared between these networks with each other (lmLdN–rmLdN vs. lmRdN–rmRdN correlations) in each area, to assess differences in the inter-modular coordination between the networks (Figure 4—figure supplement S11; Figure 5—figure supplement S2).

*Tasks 4 and 5*. The ipsilateral hypothalamus (HPT) and spinal cord (SpC) correlations made either by the left modules (lms) (HPT-lmLdN—SpC-lmLdN and HPT-lmRdN—SpC-lmRdN) or right module (HPT-rmLdN—SpC-rmLdN and HPT-rmRdN—SpC-rmRdN) were compared (a) between the gene networks (LdN vs. RdN) (Task 4), and (b) for each network, between the left and right modules (Task 5). This analysis assessed differences between (a) the LdN and RdN in each the left and right ipsilateral correlation pattern (Task 4) and (b) between the left and right ipsilateral correlation pattern for each the LdN and RdN (Task 5) (Figure 6).

*Tasks 6 and 7*. All the contralateral left HPT—right SpC correlations (Contralateral type 1 pattern: HPT-lmLdN—SpC-rmLdN and HPT-lmRdN—SpC-rmRdN), and the contralateral right HPT—left SpC correlations (Contralateral type 2 pattern: HPT-rmLdN—SpC-lmLdN and HPT-rmRdN—SpC-lmRdN) were compared between the LdN and RdN (Task 6) and between the Contralateral type 1 and Contralateral type 2 patterns for each LdN and RdN (Task 7) (Figure 6—figure supplement S1).

In each of 7 tasks, all correlation patterns were compared between the UBI and sham surgery groups.

## Results

### The Brain Injury-Induced HL-PA in Rats With Transected Cervical Spinal Cord

The asymmetric effects of UBI on hindlimb posture and reflexes are mediated through the descending neural tracts and the humoral pathway by the T-NES.^6^,^12^ In addition to these pathways, the top-down asymmetric signaling could be mediated by the preganglionic sympathetic neurons in the superior thoracic segments that project to the paravertebral sympathetic chain and by the postganglionic fibers that project to the hindlimbs.^60–62^ To explore this possibility, hindlimb responses to UBI were analyzed in rats with a complete spinal cord transection performed at the level rostral to the preganglionic sympathetic neurons. A 3–4-mm segment of the C6-C7 spinal cord was excised, the hindlimb representation area of the sensorimotor cortex was then unilaterally ablated by suction, and HL-PA was analyzed.

Brain lesions extended 4.8–5.6 mm rostrocaudally and 2.6–3.1 mm mediolaterally. The lesion depth was 1.0–1.5 mm and did not involve the subcortical white matter (Figure 1—figure supplement S1). Cortical lesion volumes (mean ± SD) without correction for tissue shrinkage due to fixation were similar in left (7.9 ± 2.3 mm^3^, *n* = 5) and right (7.8 ± 2.5 mm^3^, *n* = 5) UBI rats (*P* = .92; 2-tailed *t*-test).

**Figure 1.**
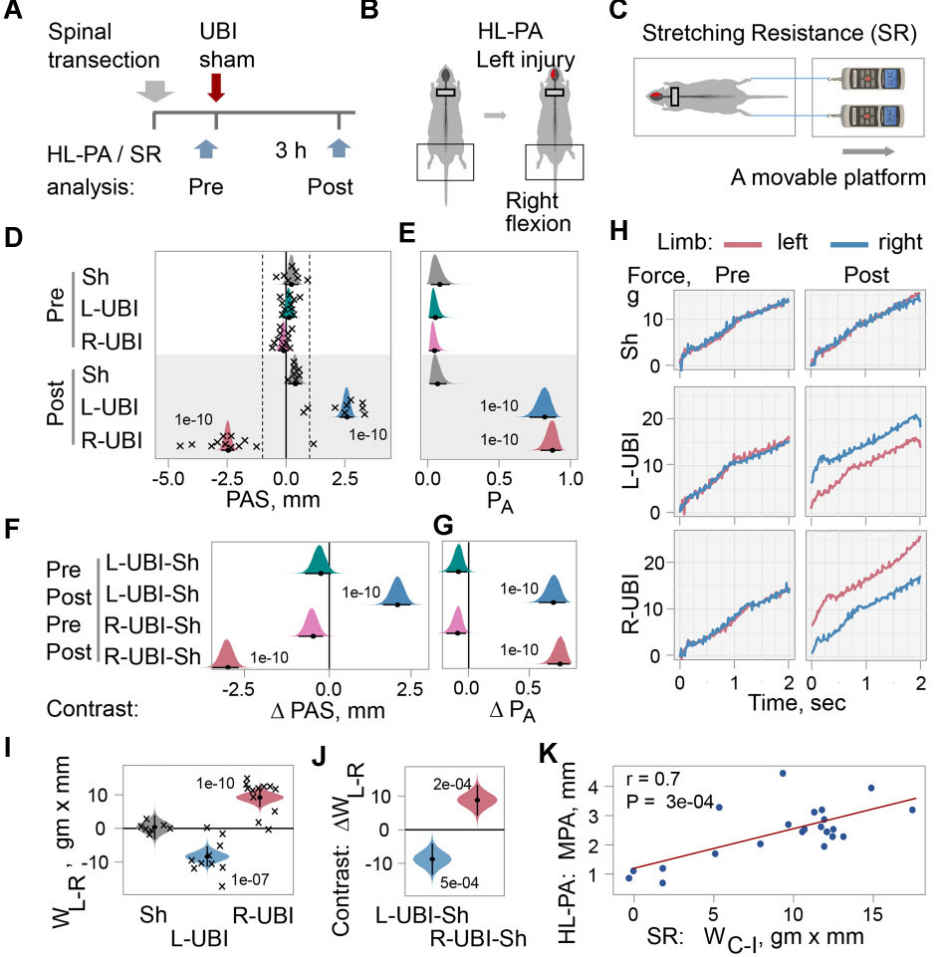
Asymmetry in hindlimb posture (HL-PA) and stretching resistance (SR) induced by the unilateral ablation of the hindlimb representation area of sensorimotor cortex (UBI) in rats with completely transected cervical spinal cords. (A) Experimental design. The spinal cord was transected at the C6-7 level and then followed by either a left UBI (L-UBI; *n* = 10), right UBI (R-UBI; *n* = 12), or sham surgery (Sh; *n* = 7). The asymmetry levels were measured before (pre) and 3 h after (post) UBI or sham surgery. (B) The UBI-induced HL-PA was manifested as flexion of the left or right hindlimb. (C) The stretching resistance was analyzed as the amount of mechanical work *W* required to stretch a hindlimb, calculated as the integral of stretching force over a distance of 0–10 mm. The force was measured using a micromanipulator-controlled force meter consisting of 2 digital force gauges fixed on a movable platform. (D) The size of postural asymmetry (PAS) measured in millimeters (mm), and (E) the probability to develop HL-PA (*P*_A_) above a 1 mm threshold (indicated in D by vertical dotted lines). Negative and positive PAS values are assigned to rats with left and right hindlimb flexion, respectively. (F and G) The contrasts in PAS and *P*_A_ between the UBI groups and the sham surgery group are denoted as ΔPAS and Δ*P*_A_, respectively, and were computed for the pre and post time points separately. (H) Representative traces of the stretching force recorded from the left and right hindlimbs before the UBI and sham surgery and 3 h after these surgeries. (I) Differences in stretching force between the left and right hindlimbs *W*_L-R_ in gm × mm analyzed 3 h after UBI or sham surgery. (J) The contrast between animal groups in *W*_L-R_ denoted as Δ*W*_L-R_ 3 h after UBI or sham surgery. (K) Pearson correlation between the magnitude of postural asymmetry (MPA) and the difference in work between the hindlimbs contralateral and ipsilateral to UBI and expressed as Δ*W*_C-I_ in gm × mm. The data presented are for left and right UBI groups analyzed 3 h after brain surgery. The median (represented as circles), 95% HPD (lines), and posterior density (distribution) from Bayesian regression are used to plot the PAS, *P*_A_, *W*_L-R_, and contrasts. Asymmetry and contrasts among the groups were deemed significant, with a 95% HPD not encompassing zero and adjusted *P*-values of ≤.05. Adjusted *P*-values are presented numerically on the plots. The PAS and *W*_L-R_ values for individual rats are indicated by crosses in D and I. Source data: The EXCEL source data file “masterfile-210807.xlsx” and source data folder “/HL-PA/data/SF/.”

#### Analysis of HL-PA

HL-PA was analyzed before (referred to as pre) and 3 h after UBI or sham surgery (post) (Figure 1A and B) using both the hands-on and hands-off methods of hindlimb stretching followed by photographic and/or visual recording of asymmetry in animals under pentobarbital anesthesia.^11^,^12^ Data from these 2 methods correlate well with each other (Figure 1—figure supplement S2). The HL-PA data presented in Figure 1 and throughout the paper are for the hands-off assay. HL-PA was characterized by (i) the size of postural asymmetry (PAS) in mm, (ii) the MPA in mm, and (iii) the probability of developing HL-PA (*P*_A_). In contrast to the MPA, the PAS shows the direction of the asymmetry; negative and positive PAS values are assigned to left and right hindlimb flexion, respectively. In the *P*_A_ calculations, rats with MPA > 1 mm were defined as asymmetric; the 1 mm MPA was the 94th percentile in rats before UBI or sham surgery and after sham surgery.

Figure 1D and E shows the middle value of each group (median), a 95% highest posterior density credible interval (95% HPD), and the probability distribution of the middle value (posterior distribution) based on Bayesian regression (see Glossary). 95% HPD is an interval within which an unobserved parameter value falls with a 95% probability. It is analogous to confidence intervals. Animals were defined as significantly asymmetric if 95% HPD in a group did not include zero and adjusted *P*-values were <.05.

Following UBI, but not sham surgery, rats with transected cervical spinal cords exhibited robust HL-PA with high statistical significance for both PAS and *P*_A_ (Figure 1D and E). Hindlimb responses to brain injury were developed on the contralesional side; thus, left UBI induced right hindlimb flexion, and right UBI induced left hindlimb flexion. The PAS and *P*_A_ in the UBI rats with cervical spinal cord transection were similar to those previously reported for UBI animals with intact and transected thoracic spinal cords.^12^,^13^ Figure 1F and G shows the contrasts between animal groups for PAS and *P*_A_, denoted as ∆PAS and ∆*P*_A_, respectively, along with the statistical significance for these contrasts (see Glossary). Each contrast is a difference between the median of the UBI group and the median of the sham surgery group. For example, the contrast ∆PAS for “Post: L-UBI-Sh” is the median of the left UBI group minus the median of the sham surgery group for PAS measured 3 h after brain surgery (post), and its *P*-value = 1e-10 after multiple correction. The PAS and *P*_A_ of left and right UBI rats were significantly greater than those of sham surgery rats 3 h after brain surgery. Both *P*_A_ and MPA did not differ between the left and right UBI groups, respectively.

#### Hindlimb Stretching Resistance

Next, we examined the effects of UBI in rats with transected cervical spinal cords on the biomechanical properties of the contra- and ipsilesional hindlimbs (Figure 1C, H–J). Hindlimb passive musculo-articular resistance to stretch was assessed in anesthetized rats before and 3 h after UBI or sham surgery. Left-right asymmetry in resistance was assessed as (i) the difference in work between the left and right hindlimbs as *W*_L-R_ = (*W*_L_ – *W*_R_), where *W*_L_ and *W*_R_ were the work applied to stretch the left and right hindlimbs, respectively (Figure 1I); and (ii) the left-right asymmetry index for work as AI_L/R_ = log_2_ (*W*_L_/*W*_R_) (Figure 1—figure supplement S3). Both the *W*_L-R_ and the AI_L/R_ were analyzed because they may depend differently on the stretching distance. The contralesional–ipsilesional asymmetry was analyzed as the difference in work applied to stretch the contralesional (C) and ipsilesional (I) hindlimb *W*_C-I_ = (*W*_C_ – *W*_I_) (Figure 1K).

Representative traces of the stretching force recorded from the left and right hindlimbs of rats with transected cervical spinal cords before and 3 h after UBI or sham surgery are shown in Figure 1H. The force required to stretch the hindlimbs increased with the degree of stretch. No significant differences were observed in stretching force between the hindlimbs in rats analyzed before sham surgery and UBI, and in rats after sham surgery (Figure 1H and I). The difference in the work required to stretch the left and right hindlimbs was strong and statistically significant 3 h after both left and right UBI, while no asymmetry was evident after sham surgery (Figure 1I; Figure 1—figure supplement S3B). The contrasts between both the left and right UBI groups and the sham surgery group in *W*_L-R_ (Figure 1J) and AI_L/R_ (Figure 1—figure supplement S3C) were robust and statistically significant. Both left and right UBI increased resistance to stretching of the contralesional hindlimb compared to the ipsilesional hindlimb. The stretching resistance’s *W*_C-I_ strongly correlated with MPA (Figure 1K).

We concluded that the hindlimb asymmetries induced by UBI were not mediated by the sympathetic nervous system or descending neural tracts. Instead, the humoral pathway may be the sole option for left-right side-specific signaling from the injured brain to hindlimb muscles in these experiments.

### Effects of Bilateral Deafferentation of Lumbar Spinal Segments on HL-PA Induced by the Left UBI or Right UBI

#### HL-PA Formation

**Figure 2.**
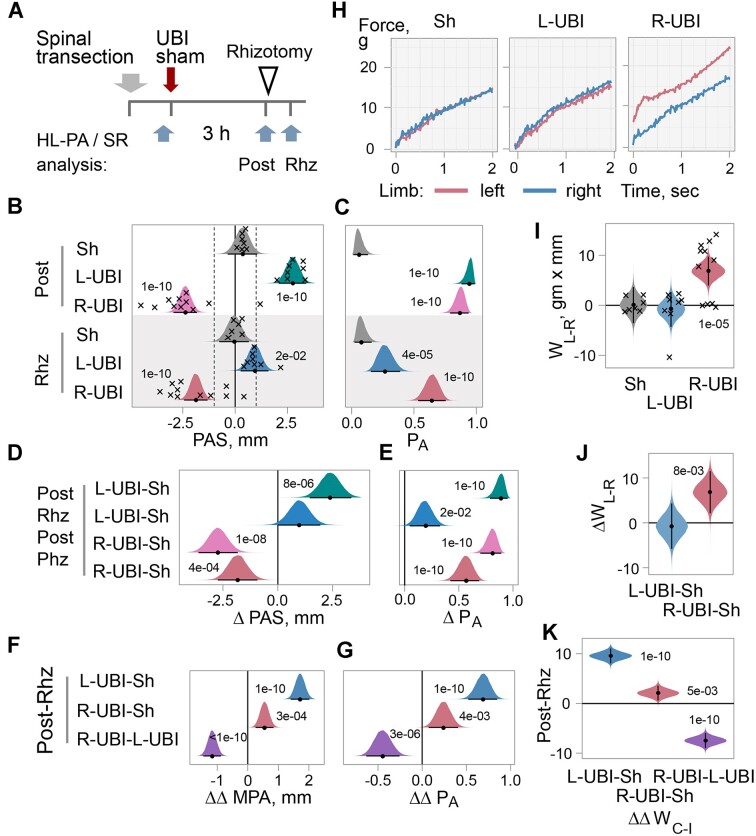
Effects of bilateral deafferentation of lumbar spinal cord on HL-PA and hindlimb asymmetry in stretching resistance (SR) induced by left UBI (L-UBI) and right UBI (R-UBI) in rats with completely transected cervical spinal cords. (A) Experimental design. The spinal cord was transected at the C6-7 level, followed by either a left UBI (*n* = 8), right UBI (*n* = 11), or sham surgery (Sh; *n* = 7). The asymmetries were analyzed 3 h after UBI or sham surgery (post) and in the same rats after bilateral rhizotomy performed from the L1 to S2 spinal levels (Rhz). (B) The HL-PA size (PAS) in millimeters (mm) and (C) the probability to develop HL-PA (*P*_A_) above the 1 mm threshold (denoted in B by dotted vertical lines). (D and E) The contrasts in PAS and *P*_A_ between the UBI groups and the sham surgery group are denoted as ΔPAS and Δ*P*_A_, respectively, and computed for the post and Rhz time points separately. (F and G) The effects of rhizotomy on differences in the MPA (or *P*_A_) between L-UBI, R-UBI, and sham surgery (Sh) analyzed as contrast of contrasts between (i) L-UBI and sham surgery: ΔΔMPA (or ΔΔ*P*_A_) = [(L-UBI_Post_ – Sh_Post_) – (L-UBI_Rhz_ – Sh_Rhz_)]; (ii) R-UBI and sham surgery: ΔΔMPA (or ΔΔ*P*_A_) = [(R-UBI_Post_ – Sh_Post_) – (R-UBI_Rhz_ – Sh_Rhz_)]; and (iii) R-UBI and L-UBI: ΔΔMPA (or ΔΔ*P*_A_) = [(R-UBI_Post_ – L-UBI_Post_) – (R-UBI_Rhz_ – L-UBI_Rhz_)]. (H) Representative traces of the stretching force recorded from the left and right hindlimbs of rats with UBI or sham surgery after rhizotomy. (I) Differences in stretching force between the left and right hindlimbs *W*_L-R_ in gm × mm in rats with UBI or sham surgery after rhizotomy. (J) The contrasts in *W*_L-R_ between the UBI and sham surgery groups denoted as Δ*W*_L-R_ in rats after rhizotomy. (K) The effects of rhizotomy on differences between contralesional hindlimb and ipsilesional hindlimb *W*_C-I_ = (*W*_Contra_ – *W*_Ipsi_) analyzed as contrast of contrasts between (i) L-UBI and sham surgery: ΔΔ*W*_C-I_ = [(L-UBI_Post_ – Sh_Post_) – (L-UBI_Rhz_ – Sh_Rhz_)]; (ii) R-UBI and sham surgery: ΔΔ*W*_C-I_ = [(R-UBI_Post_ – Sh_Post_) – (R-UBI_Rhz_ – Sh_Rhz_)]; and (iii) R-UBI and L-UBI: ΔΔ*W*_C-I_ = [(R-UBI_Post_ – L-UBI_Post_) – (R-UBI_Rhz_ – L-UBI_Rhz_)]. The PAS, *P*_A_, MPA, *W*_L-R_, *W*_C-I_, and contrasts are plotted as median (circles), 95% HPD (lines), and posterior density (distribution) from Bayesian regression. Asymmetry and contrasts among the groups were deemed significant, with a 95% HPD not encompassing zero and adjusted *P*-values of ≤.05. Adjusted *P-*values are presented numerically on the plots. Crosses in (B) and (I) denote the PAS and *W*_L-R_ values for individual rats, respectively. Source data: The EXCEL source data file “masterfile-210807.xlsx” and source data folder “/HL-PA/data/SF/.”

HL-PA may develop in response to activation of spinal reflexes or changes in spinal circuits.^11^ We next sought to determine whether afferent somatosensory input is required for persistence of HL-PA induced by the UBI through a humoral pathway. Left or right UBI was performed in rats with transected cervical spinal cords, and the effects of bilateral rhizotomy of the dorsal roots from the L1 to S2 levels on HL-PA were analyzed (Figure 2). In rats with left UBI, the PAS and *P*_A_ were markedly reduced, 3.0- and 3.5-fold, respectively, after rhizotomy (Figure 2B and C). Contrariwise, in the right-side UBI rats, the PAS and *P*_A_ demonstrated only small, approximately 1.3-fold, decreases after rhizotomy. No signs of asymmetry were revealed in the sham surgery rats after rhizotomy.

Contrast in both the PAS and *P*_A_ was strong and highly significant between the UBI groups and the sham surgery group before the rhizotomy and between the right UBI and sham surgery group after rhizotomy (Figure 2D and E). The left UBI minus sham surgery contrast was negligible in rats analyzed after rhizotomy. The relative impact of rhizotomy on the effects of left and right UBI was analyzed as contrast of contrasts (Figure 2F and G). Each contrast of contrasts is a difference between the contrast before rhizotomy minus the contrast after rhizotomy. For example, the contrast of contrasts ∆∆MPA for “Post-Rhz: L-UBI-Sh” (Figure 2F) is the contrast ∆MPA “Post: L-UBI-Sh” measured before rhizotomy (Figure 2D) minus the contrast ∆MPA “Rzh: L-UBI-Sh” measured after rhizotomy (Figure 2D), and *P*-value = 1e-10 for this ∆∆MPA after multiple correction. The contrast of contrasts was high and significant when comparing the left UBI group to the sham surgery group, whereas it was much smaller when the right UBI rats were compared with sham rats. Most interesting, contrast of contrasts was high and significant for comparison of the right UBI group to the left UBI group (Figure 2F and G).

#### Stretching Resistance Analysis

The stretching resistance of the contra- and ipsilesional hindlimbs in rats with transected cervical spinal cords was analyzed before and after the bilateral rhizotomy that was performed 3 h after UBI or sham surgery (Figure 2H–K; Figure 2—figure supplement S1). In rats with left UBI but not with right UBI, rhizotomy abolished the differences in resistance between the hindlimbs (Figure 2I; Figure 2—figure supplement S1B). No rhizotomy effects were evident in rats with sham surgery. Whereas contrast in both the *W*_L-R_ and AI_L/R_ between UBI groups and sham surgery group was strong and highly significant before rhizotomy (Figure 2J), no differences were evident between left UBI group and sham surgery group after rhizotomy (Figure 2J; Figure 2—figure supplement S1C). Contrasts between the right UBI group and the sham surgery group remained strong and significant after rhizotomy.

Impact of rhizotomy (contrast: before vs. after rhizotomy) on the effects of left and right UBI (contrast: UBI vs. sham surgery) was compared as contrast of contrasts in both the Δ*W*_CI_ and ΔAI_CI_ (Figure 2K; Figure 2—figure supplement S1D). The contrast of contrasts for the left UBI group was high and significant, while that of the right UBI group was noticeably smaller. Furthermore, the effects of left and right UBI in both the *W*_C-I_ and AI_C-I_ were differently affected by rhizotomy as evident from the analysis of contrast of contrasts (Figure 2K; Figure 2—figure supplement S1).

Thus, the HL-PA and stretching resistance data correlate with each other and demonstrate that the effects of the left and right UBI mediated through the humoral pathway are differentially sensitive to bilateral deafferentation. The effects of the left UBI but not the right-side UBI apparently depend on afferent somatosensory input. These results align with previous findings that in rats maintained for 3 d with intact spinal cords after UBI, bilateral lumbar dorsal rhizotomy eliminated HL-PA after the left but not the right lesion.^11^ The experiments were performed under isoflurane anesthesia. Hence, the sidedness of the effects of deafferentation does not depend on whether the spinal cord is transected before or after brain injury and on the type of anesthesia (ie, isoflurane or pentobarbital).

### Effect of Opioid Antagonists on Hindlimb Asymmetry in Posture and Stretching Resistance Induced by Left and Right UBI

**Figure 3.**
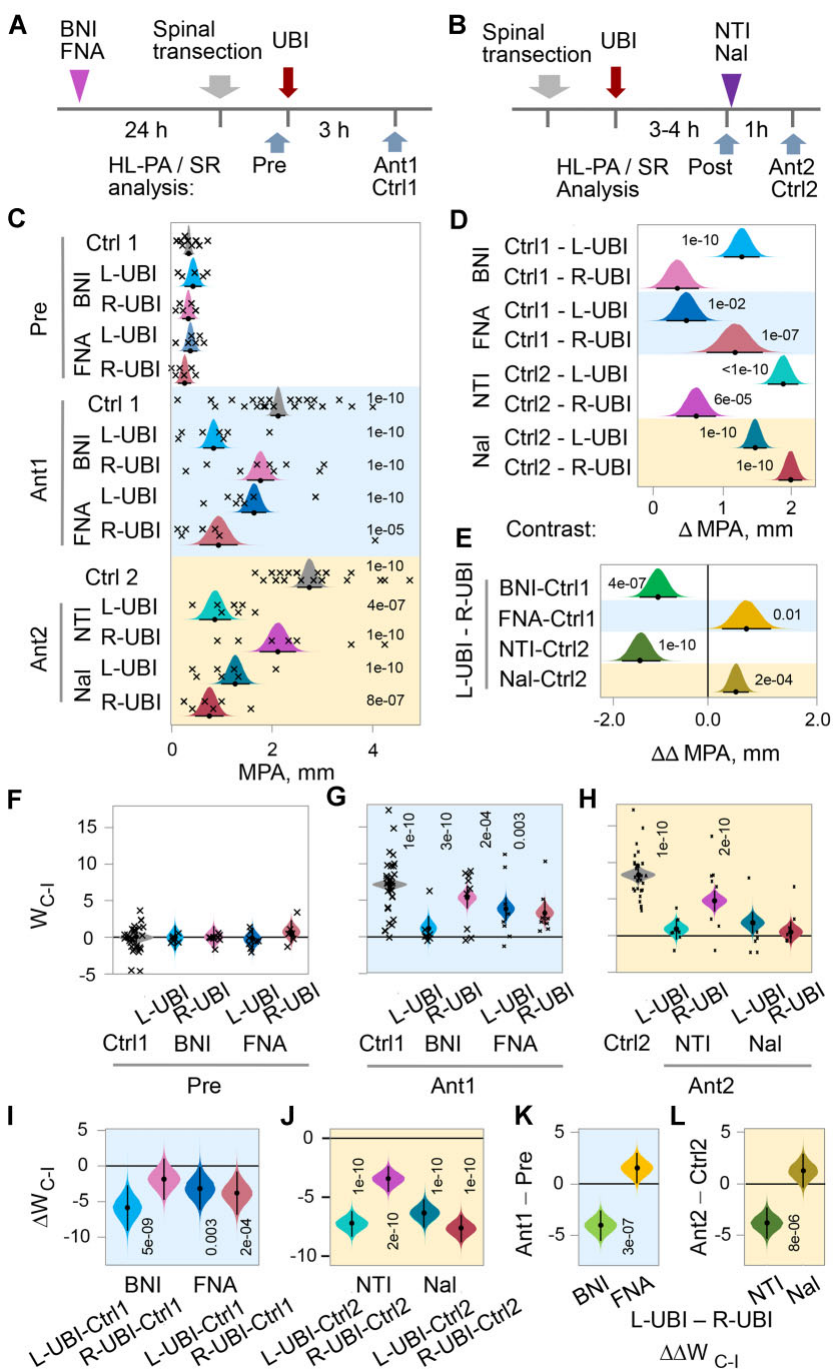
Effects of nor-binaltorphimine (BNI), β-funaltrexamine (FNA), and naltrindole (NTI), the selective κ-, µ-, and δ-opioid antagonists, respectively, and naloxone (Nal), the general opioid antagonist, on HL-PA and hindlimb asymmetry in stretching resistance (SR) induced by left UBI (L-UBI) and right UBI (R-UBI) in rats with completely transected cervical spinal cords. The spinal cord was transected at the C6-7 level, and then left or right UBI was performed. (A) Experimental design 1. BNI and FNA were administered 24 h before the transection (Ant1). The asymmetries were analyzed after the transection before the UBI (pre), and then 3 h after the UBI (Ant1). The control group Ctrl1 consisted of rats with UBI that were not treated with drugs. (B) Experimental design 2. The asymmetries were assessed 3 h after UBI (Post). The rats with MPA greater than 1.5 mm were selected for further analysis and treated with NTI or Nal (Ant2) or used as controls after saline treatment, or left untreated (Ctrl2). Asymmetries were analyzed 1 h later. Group design and the number of rats in the groups are given in Figure 3—figure supplement S1. The direction of PAS in all animals in each group was the same; the left and right UBI rats exhibited positive and negative PAS values, respectively. (C) The antagonist effects on the MPA. (D) Contrasts in the MPA between the respective control groups and the groups treated with antagonists. Time points: 3 h after transection for BNI and FNA (Ant1), and 4 h after transection for NTI and Nal, which was 1 h after their administration (Ant2). (E) Contrast of contrasts between the L-UBI and R-UBI groups in the effects of antagonists on the MPA: ΔΔMPA = [(L-UBI_Ant1_ – Ctrl1) – (R-UBI_Ant1_ – Ctrl1)] for BNI and FNA; and ΔΔMPA = [(L-UBI_Ant2_ – Ctrl2) – (R-UBI_Ant2_ – Ctrl2)] for NTI and Nal. The time points are the same as those in the (D). (F–H) Differences in stretching force between the contra- and ipsilesional hindlimbs *W*_C-I_ in gm × mm. (I and J) Contrasts in the *W*_C-I_ between the UBI groups treated with the antagonists and respective control groups. The time points are the same as those in (D). (K and L) Contrast of contrasts between the L-UBI and R-UBI groups in the effects of antagonists on the *W*_C-I_; K: ΔΔ*W*_C-I_ = [(L-UBI_Ant1_ – Pre1) – (R-UBI_Ant1_ – Pre1)] and L: ΔΔ*W*_C-I_ = [(L-UBI_Ant2_ – Ctrl2) – (R-UBI_Ant2_ – Ctrl2)]. Crosses denote the MPA and the *W*_C-I_ values for individual rats. The median (represented as black circles), 95% HPD (black lines), and posterior density (colored distribution) from Bayesian regression are used to plot the MPA, *W*_C-I_, and contrasts. Asymmetry and contrasts among the groups were deemed significant, with a 95% HPD not encompassing zero and adjusted *P*-values of ≤.05. Adjusted *P*-values are presented numerically on the plots. Source data: the EXCEL source data file “SDU-RDPA-Stat_v2.xlsx” and source data folder/HL-PA-opioid-antagonists/data/SF/.”

We previously demonstrated that the selective opioid antagonists inhibited the formation of HL-PA after UBI in rats with intact spinal cords and that the effects of the antagonists were left-right side-specific.^13^ These findings suggest that the opioid system controls signaling from the injured area to spinal motoneurons through descending neural tracts and that the antagonists interfere with this process. However, the asymmetric effects of UBI on hindlimb posture also were blocked by naloxone, a non-selective opioid antagonist, in rats with transected spinal cords indicating the involvement of the opioid receptors in neuroendocrine signaling from the injured brain through the bloodstream.^12^ Here, we examined whether the opioid system controls UBI effects mediated by the humoral pathway and determined whether this control is side- and receptor-subtype-specific. The effects of the selective µ-, δ-, and κ-opioid antagonists β-funaltrexamine (FNA), naltrindole (NTI), and nor-binaltorphimine (BNI), respectively, and naloxone on the asymmetry in hindlimb posture and stretching resistance in rats with completely transected cervical spinal cords were compared between the left and right UBI groups (Figure 3; Figure 3—figure supplement S1). BNI and FNA are long-acting antagonists that selectively block their receptor subtypes after approximately 24 h.^30–33^ The rats were administered with these antagonists 24 h before UBI (Figure 3A). NTI and naloxone were administered 3 h after UBI to rats that exhibited HL-PA with the MPA > 1.5 mm. HL-PA was then analyzed 1 h later (Figure 3B).

In the left UBI group, there was a significant reduction in MPA by 3.2-fold induced by NTI and 2.5-fold induced by BNI, while the FNA effects were not pronounced (Figure 3C and D). In contrast, in the right-side UBI group administration of FNA, but not of NTI or BNI, resulted in substantial MPA reduction (2.3-fold). Data were analyzed for the MPA in order to compare the effects of opioid antagonists on HL-PA after left UBI and right UBI, which was not feasible with the PAS. Naloxone inhibited the effects of both the left- and right-side injuries (2.2- and 3.7-fold, respectively). The effects of the antagonists were significantly different between the left and right UBI groups (Figure 3E). NTI and BNI preferentially inhibited formation of the right hindlimb flexion, whereas, in contrast, FNA inhibited flexion on the left side.

Administration of BNI and NTI markedly decreased the contra-ipsilesional hindlimb asymmetry in stretching resistance *W*_C-I_ after the left UBI, while their effects were minor in rats with the right UBI (Figure 3F–L). The FNA effects were less pronounced (Figure 3G, I, and K). Naloxone substantially reduced the *W*_C-I_ in rats either with the left or right UBI (Figure 3H, J, and L). Analysis of contrast of contrasts revealed significant differences between the left and right UBI groups in the effects of BNI and NTI (Figure 3K and L). The effect of FNA on the *W*_C-I_ after the right UBI slightly exceeds that after the left side injury (Figure 3K).

Thus, the HL-PA and stretching resistance data are in general agreement; the effects of the left and right UBI were differently inhibited by the opioid antagonists suggesting that the left and right T-NES counterparts are differentially controlled by the opioid receptor subtypes.

### Gene Expression and Co-Expression Patterns

Signals from the injured hemisphere may be encoded into left-right side-specific hormonal messages in the hypothalamic–pituitary system that then are released into the blood.^12^ These messages target the lumbar spinal cord and may produce lateralized changes in gene expression. As a result, the UBI-induced changes in the balance between the left and right gene expression patterns may be coordinated across the neuroendocrine and motor regions.

The prerequisite for the left-right side-specific encoding of neurohormonal messages may be an asymmetrical organization of hypothalamic neurosecretory circuits, including their gene expression profiles, and the side-specific responsiveness of these circuits to a unilateral impact. We previously reported that the “decoding” lumbar spinal cord is characterized by asymmetric gene expression patterns and that the UBI produced the ipsi-contralesional side-specific changes in gene expression and gene–gene co-expression in rats with complete spinal cord transection.^12^,^13^,^63^

Here, we examined if UBI targets the hypothalamus and pituitary gland as the “encoding” areas by analysis of gene expression; i.e., if UBI affects gene expression in the ipsi- or contralesional hypothalamus; and if hypothalamic expression of neurohormonal and neuroplasticity-related genes is lateralized. To reveal regulatory humoral interactions between the “encoding” hypothalamus and “decoding” spinal cord, we then characterized gene–gene co-expression patterns between these regions along with perturbations in these patterns produced by UBI in rats with complete spinal cord transection. Tissue samples were collected 3 h after left UBI or left sham surgery that was performed in rats with completely transected thoracic spinal cord. Expression data for the lumbar spinal cord of these rats were taken from our previous study.^12^ The left UBI was used because its effects were stronger than those of the right-side injury in rats with intact spinal cords^11^,^13^ and because it produced changes in left-right coordination of expression of the neuropeptide and neuroplasticity-related genes in the lumbar spinal cord of rats with transected spinal cords.^12^

#### The Hypothalamic–Pituitary System

We reasoned that the “encoding” system that mediates the neuroendocrine UBI effects in the hypothalamus involves genes of the releasing and inhibitory hormones (*Crh, Ghrh, Gnrh1, Sst*, and *Trh*), neuropeptides and their receptors genes (*Avp, Avpr1a, Nts, Penk, Pdyn, Pomc, Oprm1, Oprk1, Oprd1 *, and *Oxt*; Figure 4—figure supplement S1-S3), along with neuroplasticity-related genes coding for regulators of axonal sprouting, synapse formation, neuronal survival, and neuroinflammation (*Arc, Bdnf, Dlg4, Homer-1, Gap43, Syt4*, and *Tgfb1*), transcriptional regulators of synaptic plasticity (*cFos, Egr1*, and *Nfkbia*), and essential components of the glutamate system critical for neuroplasticity (*GluR1, Grin2a*, and *Grin2b*) (Figure 4—figure supplement S4). These genes were revealed as neuroplasticity-related in several studies each (for details of gene selection, see the “Materials and methods” section).

**Figure 4.**
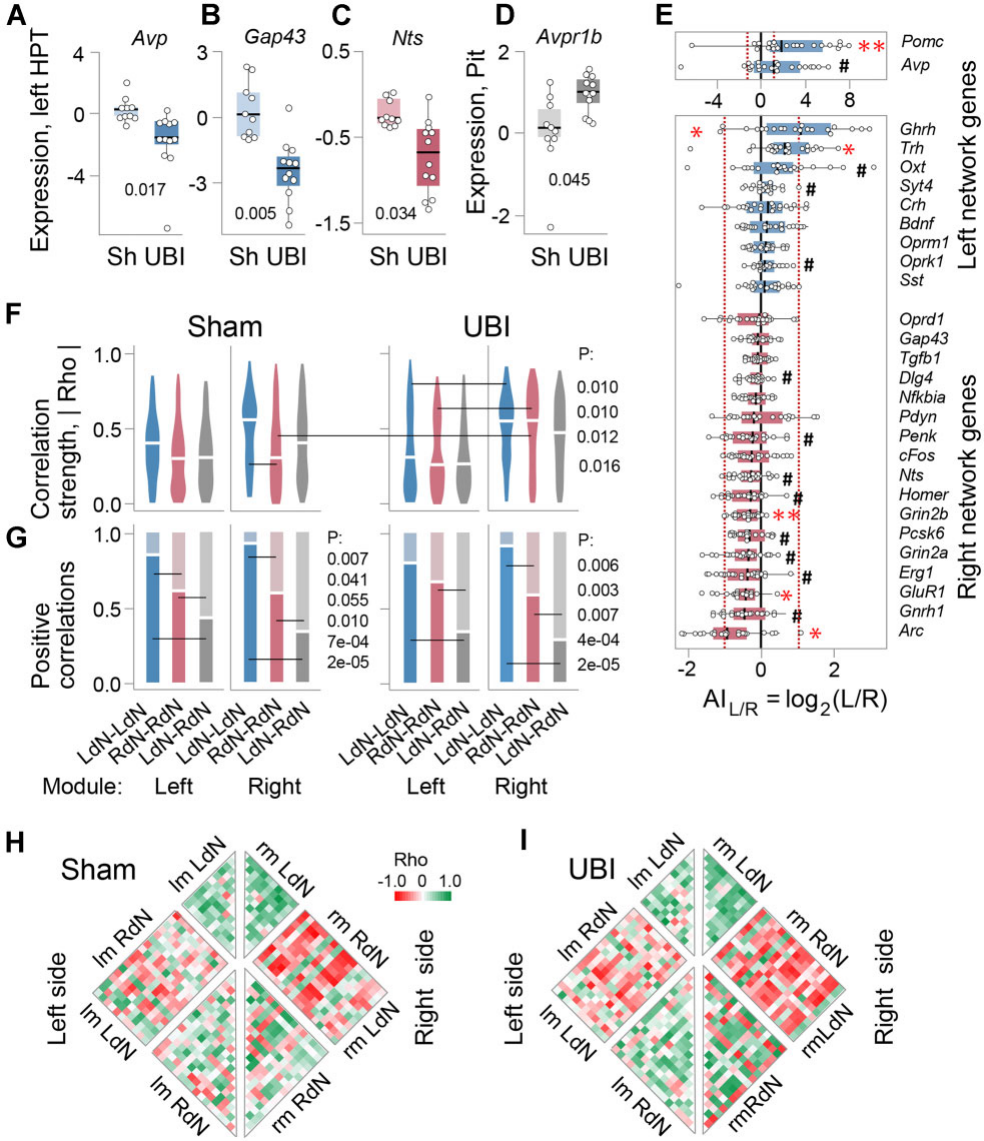
The UBI effects on gene expression patterns in the hypothalamus and pituitary gland. Analysis of the left (LdN) and right (RdN) dominant gene co-expression networks in the hypothalamus. (A–D) Gene expression levels in the left hypothalamus (HPT) and the pituitary gland (Pit) collected 3 h after left sham surgery (Sh: *n* = 11 rats) or left UBI (*n* = 12 rats) that were performed in the spinalized rats. The expression levels in the log_2_ scale and the Bonferroni adjusted *P*-values determined by Mann–Whitney test are shown. (E) The asymmetry index AI_L/R _= log_2_[L/R], where L and R are the median expression levels in the left and right hypothalamus, is shown for each gene analyzed. There were no differences in the AI_L/R_ between sham surgery and UBI groups; therefore, they were combined (*n* = 22) for statistical analysis. Wilcoxon signed-rank test followed by Bonferroni multiple testing correction: *, *P-*adj < .05; **, *P-*adj < .01; #, *P* ≤ .05 (not adjusted). Boxes denote genes with AI_L/R _> 0 or AI_L/R _< 0 that were defined as the LdN and RdN genes. In (A–E), data are presented as boxplots with median and hinges representing the first and third quartiles, and whiskers extending from the hinge to the highest/lowest value that lies within the 1.5 interquartile range of the hinge. (F and G) Patterns of intra-modular correlations internal for each the LdN (LdN–LdN) and RdN (RdN–RdN), and between the networks (LdN–RdN) are shown for the left and right modules of sham surgery and left UBI groups. In (F), correlation strengths |Rho| (absolute value of each pairwise correlation) are presented as violin plots with white line indicating mean coordination strength. The proportion of positive correlations is shown as horizontal lines in (G). The 3 correlation patterns were compared within each (ie, left and right) module. Each pattern was compared between the modules and between UBI and sham surgery groups (for details, see Figure 4—figure supplement S10A). *P*-values were determined by permutation testing with Benjamini–Hochberg family wise multiple test correction. Significance for contrasts was determined by analysis of 3 AI_L/R_ categorization variants (Figure 4—figure supplement S9); *P*-values are shown for the categorization variant with the median AI_L/R_ of the combined sham surgery and UBI group. (H and I) Heatmaps for Spearman’s rank coefficients for pairwise gene–gene correlations in the left- (lm) and right- (rm) modules of sham surgery and UBI groups. Source data: the EXCEL source data files “Hypoth_SO_UBI.xlsx; RD Hypophis_ Master file.xlsx; Table III-S6 23 05 10.xlsx; raw_groups.xlsx.”

Expression of the *Avp* [fold change (FC)=3.46x], *Gap43* (FC = 1.18x), and *Nts* (FC = 1.32x) genes was significantly affected by the left UBI in the left hypothalamus (Figure 4A–C). Also, the UBI effects were nominally significant for the *Crh* (FC = 1.76x), *Sst* (FC = 1.59x), *Bdnf* (FC = 1.25x), *Syt4* (FC = 1.22x), *Pomc* (FC = 2.49x), and *Ghrh* (FC = 1.63x) genes (Figure 4—figure supplement S5). The expression levels of these genes were lower in the left hypothalamus in the UBI group compared to sham surgery group. In the right hypothalamus, expression of these genes was decreased but the UBI effects were not significant. Nonetheless, the UBI-induced changes were consistent between the left and right sides in their magnitude. Pearson and Spearman’s rank correlation coefficients between log-scaled FCs induced by the left UBI in the left and the right hypothalamus were equal to 0.79 (*P* = 5.4 × 10^−7^) and 0.48 (*P* = .010), respectively (Figure 4—figure supplement S6). Fitting the data with a linear model with an arbitrary intercept (logFC_right_ ≈ *a* logFC_left_ + *b*) resulted in estimates of *a* = 0.64 (95% CI [0.45, 0.83]) that was significantly smaller than 1. The estimate for *b* was close to zero (–0.02; 95% CI [−0.12, 0.08]). Consistently, absolute values of the FC significantly differed between the sides (Wilcoxon signed rank test: *P* = .018). Thus, the effects of left UBI were significantly greater in the left hypothalamus than in the right hypothalamus.

No significant differences in the AI_L/R _= log_2_[L/R] (where L and R denote expression levels in the left and right hypothalamus, respectively) were identified between UBI and sham groups, and these groups were combined for analysis of lateralization. Comparison of the AI_L/R_ with zero identified 3 genes (*Pomc, P* = .009; *Trh, P* = .014; and *Ghrh, P* = .014) with higher expression in the left hypothalamus while other 3 genes (*Grin2b, P* = .002; *GluR1, P* = .010; and *Arc, P* = .010) showed higher expression on the right side (Figure 4E). Lateralization was nominally significant for the *Avp, Oprk1, Syt4*, and *Oxt* genes that showed higher expression in the left hypothalamus, and for the *Grin2a, Homer, Nts, Erg1, Pcsk6, Penk, Gnrh1*, and *Dlg4* genes that demonstrated higher expression on the right side.

In the pituitary gland, the expression levels of the hormonal (*Fshb, Cga, Gh1, Lhb, Prl *, and *Tshb*), opioid peptides and their receptors (*Oprm1, Oprd1, Oprk1, Pdyn, Penk*, and *Pomc*), oxytocin (*Oxt*), Arg-vasopressin and its receptors (*Avp, Avpr1a, Avpr1b*, and *Avpr2*) (Figure 4—figure supplements S2, S3, and S7) genes were compared between the left UBI and left sham surgery groups. The expression levels of the *Avpr1b* gene were significantly elevated (FC = 1.84x) (Figure 4D), while those of the *Oxt* (FC = 1.17x) and *Tshb* (FC = 1.63x) genes (Figure 4—figure supplement S8) were increased with nominal significance in the UBI group.

Thus, in the hypothalamus expression of a subset of the neurohormonal, neuropeptide, and neuroplasticity-associated genes was lateralized and affected by UBI on the ipsilesional side. Among genes responded to UBI in the neuroendocrine system were *Avp, Avpr1b*, and *Pomc* that give rise to Arg-vasopressin, the vasopressin receptor V1B and β-endorphin that, as we have demonstrated, mediate the effects of the left UBI on HL-PA through humoral pathway.^12^

#### Gene–Gene Co-Expression within and between the Hypothalamus and Spinal Cord

Analysis of gene–gene co-expression patterns uncover regulatory interactions between tissues and brain areas.^11^,^64–67^ Here, we evaluated if such patterns are coordinated in a lateral fashion across the hypothalamus and spinal cord, and if this coordination is mediated through humoral pathway and affected by left UBI. To take into account the lateralization factor, we separately analyzed genes with higher expression either on the left- or right-side of the hypothalamus and the spinal cord, and defined them as the left dominant network (LdN; AI_L/R _> 0) or right dominant network (RdN; AI_L/R _< 0) genes in each region. We examined if gene–gene co-expression patterns differ between these networks and for both network between the sides in the hypothalamus and spinal cord; if the patterns are coordinated between these regions; and if the coordination is ipsi- or contralateral and perturbed by a unilateral brain lesion. Gene–gene co-expression was analyzed by pairwise Spearman correlations. The coordination strength and the directions (signs) of interactions in the gene–gene co-expression patterns were assessed as mean of the absolute value of the correlation coefficient Rho and the proportion of positive correlations, respectively.

All samples were dissected from the same rats with transected spinal cord that also had the left UBI or left sham surgery. Genes of the opioid and vasopressin systems were included because of their neuropeptide products are involved in asymmetric spinal responses to brain injury. The set of neuroplasticity-related genes was the same in the hypothalamus and the spinal cord (described in the “Materials and methods” section).

##### Categorization of Genes into the Left and Right Networks

**Figure 5.**
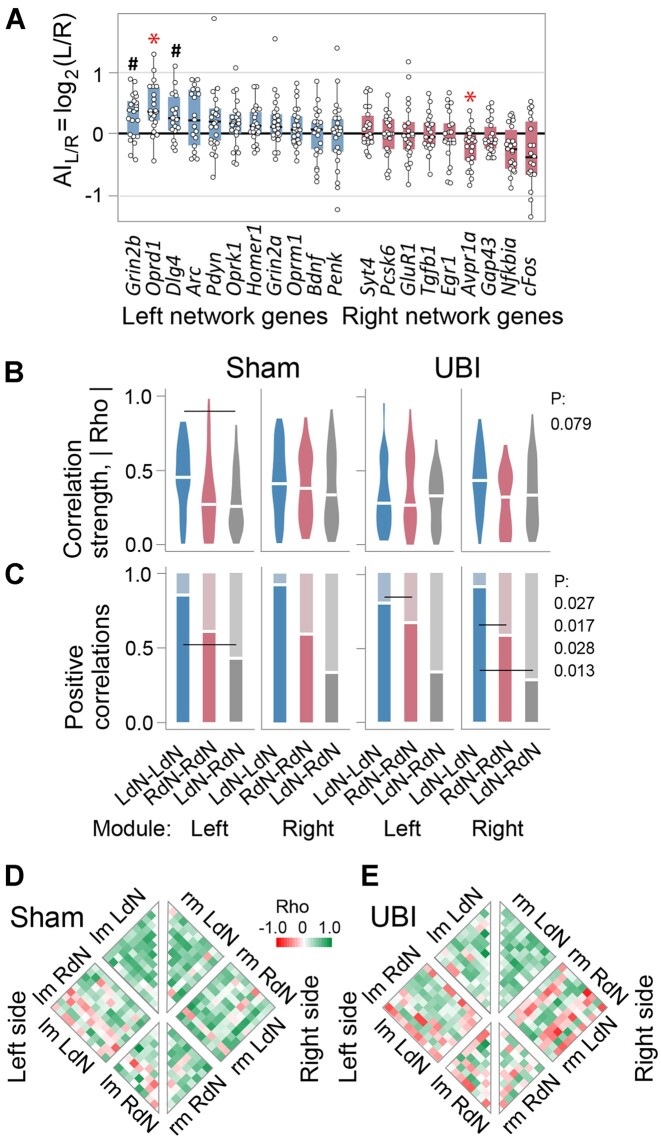
Gene co-expression patterns in the left and right lumbar spinal cord of the sham surgery and UBI rats. Analysis of the LdN and RdN gene networks. Expression of the neurohormonal and neuroplasticity-related genes was analyzed in the left and right halves of the spinal cord that were isolated 3 h after the left sham surgery (*n* = 11) or left UBI (*n* = 12) in spinalized rats. (A) Gene categorization using the AI_L/R _= log_2_[L/R], where L and R are the median expression levels in the left and right spinal cord into the LdN (AI_L/R_ > 0) and RdN (AI_L/R_ < 0). The median AI_L/R_ is shown for the combined sham surgery and the left UBI group. There were no differences in the AI_L/R_ between sham surgery and UBI groups; therefore, they were combined (*n* = 22) for statistical analysis (for details, see Figure 5—figure supplement S1). Wilcoxon signed-rank test followed by Bonferroni multiple testing correction: *, *P-*adj < .05; #, *P* ≤ .05 (not adjusted). Data are presented as boxplots with median and hinges representing the first and third quartiles, and whiskers extending from the hinge to the highest/lowest value that lies within the 1.5 interquartile range of the hinge. (B and C) Patterns of intra-modular correlations internal for each the LdN (LdN–LdN) and RdN (RdN–RdN), and between the networks (LdN–RdN). A violin plot for absolute Rho values of pairwise correlations, with horizontal line indicating overall coordination strength (defined as an average of these absolute values) in (B) and the proportion of positive correlations in (C) for the left and right modules are shown for the sham surgery and UBI groups. The 3 correlation patterns were compared within each (ie, left and right) module. Each pattern was compared between the modules, and between UBI and sham surgery groups (for details, see Figure 4—figure supplement S10B). *P*-values were determined by permutation testing with Benjamini–Hochberg family wise multiple test correction. Significance for contrasts was determined by analysis of 3 AI_L/R_ categorization variants (Figure 5—figure supplement S1); *P*-values are shown for the categorization variant with the median AI_L/R_ of the combined sham surgery and UBI group. (D and E) Heatmaps for Spearman’s rank coefficients for pairwise gene–gene correlations in the left- (lm) and right- (rm) modules of sham surgery and UBI groups. Source data: the EXCEL source data file “SpinalC_SO_UBI_Ctrl_RD_DD.xlsx; Table III-S6 23 05 10.xlsx; raw_groups.xlsx.”

Genes were categorized into the LdN and RdN in the hypothalamus and spinal cord separately. To avoid a bias, the categorization was performed in 3 variants: The LdN and RdN genes were defined by (1) their median AI_L/R_ in the combined sham surgery and UBI group; (2) their median AI_L/R_ in sham surgery group only; and (3) their mean AI_L/R_ in the combined sham surgery and UBI group (Figures 4E and 5A; Figure 4—figure supplement S9; Figure 5—figure supplement S1). A *P*-value between correlation patterns was determined by the permutation test with Benjamini–Hochberg family wise multiple test correction. The contrast was defined as significant using a stringent criterion: i.e., if the *P*-value was ≤.05 for (i) all 3 variants after correction, or for (ii) any 2 of them while for the third variant it was <.05 and <.10 before and after the correction, respectively. Left-side gene expression was defined as left module (lm) of LdN and RdN networks, whereas right-side expression as their right module (rm).

##### Correlation Patterns in the Hypothalamus and Spinal Cord

We compared the LdN and RdN in their coordination strength and the proportion of positive correlations for intra-modular correlations (LdN–LdN, RdN–RdN, and LdN–RdN) for both left and right modules separately; these intra-modular correlations between left and right modules; and inter-modular correlations between the networks (ie, lmLdN–rmLdN and lmRdN–rmRdN, respectively) (Figures 4 and 5; Figure 4—Supplements S10 and  S11; Figure 5—Supplement S2). These comparisons were performed for both the sham surgery and the UBI groups separately, and between them.

In the hypothalamus, the permutation test revealed significant differences in the coordination strength between internal LdN and RdN correlations (LdN–LdN > RdN–RdN) in the rm of sham surgery rats, and between the left and right modules for both of the networks (lm < rm) in the UBI rats (Figure 4F).

The proportion of positive correlations in the hypothalamus robustly and significantly differed between internal LdN and RdN correlations, and between them and mixed (LdN–RdN) correlations (Figure 4G). The differences were revealed at all comparisons in both modules in sham surgery group (*n* = 6) and at most of them (*n* = 5) in the UBI group. The pattern of differences was the same across the modules and animal group: the proportion for LdN–LdN correlations was significantly higher than that for RdN–RdN, and for both of them was higher than that for LdN–RdN. Furthermore, the proportion was significantly larger in the inter-modular LdN (lmLdN–rmLdN) correlations compared to the inter-modular RdN (lmRdN–rmRdN) correlations (Figure 4—figure supplement S11).

In the spinal cord, the coordination strength was significantly higher in LdN compared to a mixed pattern (LdN–RdN) in the lm of sham surgery rats (Figure 5B). The pattern of differences in the proportion of positive correlations was generally the same as that in the hypothalamus, however less contrasts were significant (Figure 5C). The proportion in LdN was larger than that in RdN and mixed LdN–RdN pattern in both sham surgery and UBI groups. Furthermore, the proportion was larger in the inter-modular LdN correlations in the sham surgery group vs. UBI group (Figure 5—figure supplement S2).

In summary, the LdN and RdN were markedly and significantly different from each other in the coordination strength and the proportion of positive correlations both in the hypothalamus and spinal cord. The UBI produced contrasting effects on the left and right hypothalamic modules in the coordination strength that were higher for both gene networks in the rm compared to the lm. For significant differences, both the coordination and the proportion were higher for the LdN compared to the RdN. Strikingly, correlations were largely positive within each network and mostly negative between the networks, suggesting positive regulatory interactions among the genes in each network and negative regulations between the networks. These differences are clearly seen on heatmaps (Figures 4H and I and 5D and E).

##### Coordination of Gene Expression between the Hypothalamus and Spinal Cord

**Figure 6.**
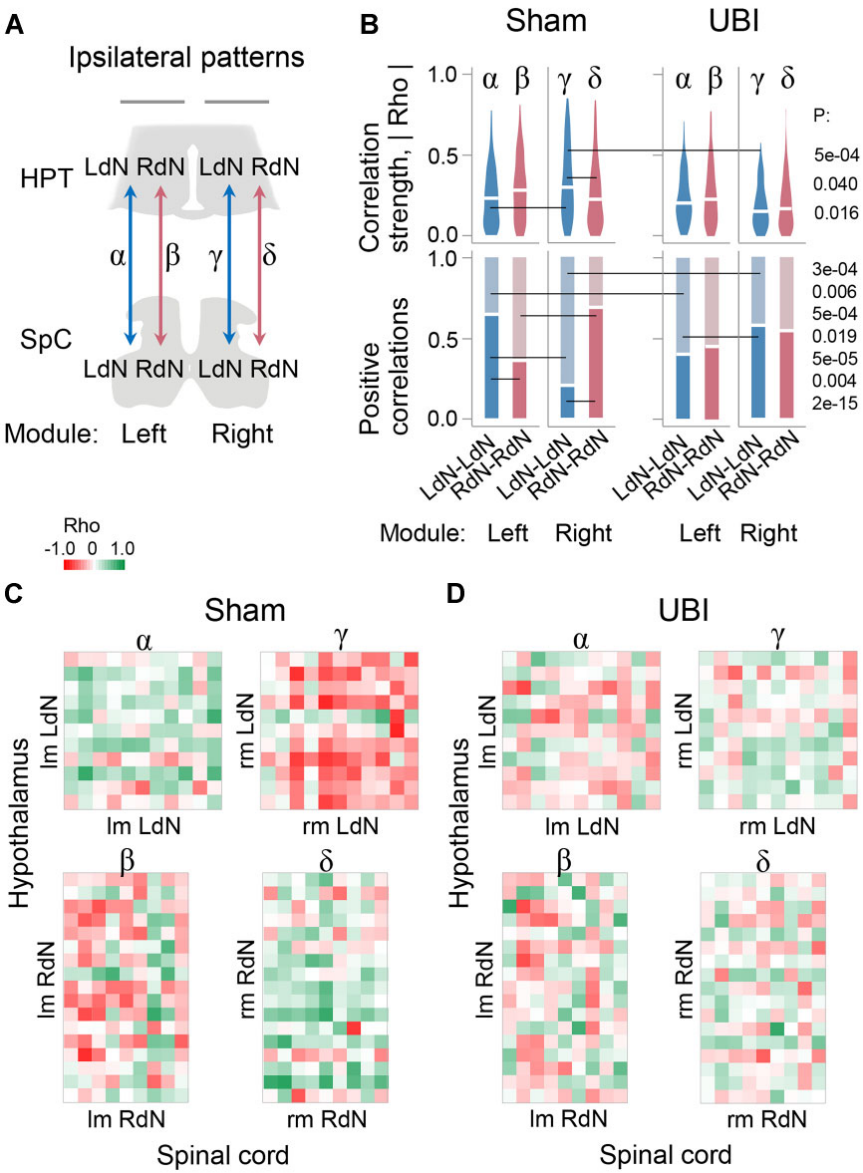
Ipsilateral coordination of the LdN and RdN between the hypothalamus and lumbar spinal cord. The effects of UBI. The experimental design and computation of LdN and RdN are described in Figures 4 and 5. (A) Analyzed patterns of the ipsilateral pairwise gene–gene Spearman rank correlations between the hypothalamus (HPT) and spinal cord (SpC) on the left side (α and β) and on the right side (γ and δ). (B) The coordination strength and the proportion of positive correlations for the correlation patterns depicted in (A). The correlation patterns were compared between the LdN and RdN (α vs. β; γ vs. δ); each of them between the left and right modules (α vs. γ; β vs. δ), and all 4 patterns individually between UBI and sham surgery groups. *P*-values were determined by permutation testing with Benjamini–Hochberg family wise multiple test correction. Significance for contrasts was determined by analysis of 3 AI_L/R_ categorization variants (Figure 4—figure supplement S9; Figure 5—figure supplement S1); *P*-values are shown for the categorization variant with the median AI_L/R_ of the combined sham surgery and UBI group. (C and D) Heatmaps for Spearman’s rank coefficients for pairwise gene–gene correlations for the left- (lm) and right- (rm) modules in sham surgery and UBI groups. Source data: the EXCEL source data file “Hypoth_SO_UBI.xlsx; SpinalC_SO_UBI_Ctrl_RD_DD.xlsx; Table III-S6 23 05 10.xlsx; raw_groups.xlsx.”

We next examined if LdN and RdN are coordinated between the hypothalamus and the lumbar spinal cord through the humoral pathway, and if this crosstalk is perturbed by UBI (Figure 6, Figure 6—figure supplement S1). We first analyzed the ipsilateral correlations internal for each LdN and RdN, between the left modules of the hypothalamus and spinal cord; and separately between their right modules (Figure 6). The LdN coordination strength in sham surgery group was asymmetric with higher level in the rm while the asymmetry was diminished after the UBI (Figure 6B). The proportion of positive correlations for both LdN and RdN in the sham surgery group was strongly asymmetric: It was much higher on the left vs. right side for the LdN, and, to the contrary, on the right vs. left side for the RdN. At the same time, the proportion and coordination strength were quite similar between LdN patterns in the left modules and RdN patterns in the right modules, and vice versa between RdN patterns in the left modules and LdN patterns in the right modules (Figure 6B and C).

Left UBI impaired the hypothalamic-spinal cord crosstalk (Figure 6B and D). Notably, only the LdN patterns were significantly affected. UBI resulted in a strong decrease of the coordination strength and elevation of the proportion of positive correlations in the LdN patterns on the contralesional, right side. Contrariwise, this proportion was strongly decreased on the ipsilesional, left side. Furthermore, in the UBI group, the proportion was higher for the right-side LdN pattern than for the left-side LdN pattern.

Analysis of the contralateral (diagonal) correlations between the left hypothalamus and right spinal cord and between the right hypothalamus and left spinal cord for each the LdN and RdN did not reveal significant patterns and UBI effects (Figure 6—figure supplement S1).

In conclusion, the robust side-specific ipsilateral patterns in the coordination of gene expression between the hypothalamus and spinal cord that differed between LdN and RdN and strong perturbations in these patterns by the unilateral ablation injury in animals with transected spinal cords were revealed. The findings suggest a functional link between these 2 regions that is the ipsilateral, left-right side-specific, and mediated by the endocrine signaling.

## Discussion

### The Left-Right Side-Specific Humoral Signaling from the Brain to the Spinal Cord: An Alternative to Neural Pathways

Our earlier study showing that HL-PA and asymmetry in hindlimb reflexes occurred in rats with completely transected spinal cords was the basis for the hypothesis that the contralateral effects of unilateral brain lesions are mediated through humoral pathway.^6^,^12^ However, signaling from the brain to the lumbar spinal cord through the paravertebral sympathetic chain was not excluded in this study because the spinal transection was performed at the T2-T3 level and the neural connections between the brain and the superior preganglionic neurons were left intact. Activity of the sympathetic preganglionic neurons located in the upper thoracic and lumbar segments is coordinated at a supraspinal (medullary) level^68^ and muscle sympathetic nerve activity is controlled by central commands.^69^ Furthermore, the spinal somato-sympathetic nerve reflexes may contribute to the maintenance of muscle contractile force both before and after spinal cord transection.^60^ In the present study, the supraspinal part of the central nervous system was fully disconnected from the preganglionic spinal neurons by spinal cord transection at the C6–C7 level that was rostral to the thoracic preganglionic sympathetic neurons. Despite the complete transection, UBI still induced asymmetric hindlimb responses. Thus, the mechanisms of the brain injury-induced HL-PA formation mediated through both the paravertebral sympathetic ganglia and descending neural pathways were ruled out. These experiments presented unambiguous proof of the left-right side-specific endocrine signaling in rats with transected spinal cords.

### The Bipartite T-NES: Intrinsic Neurohormonal and Neural Asymmetry

**Figure 7.**
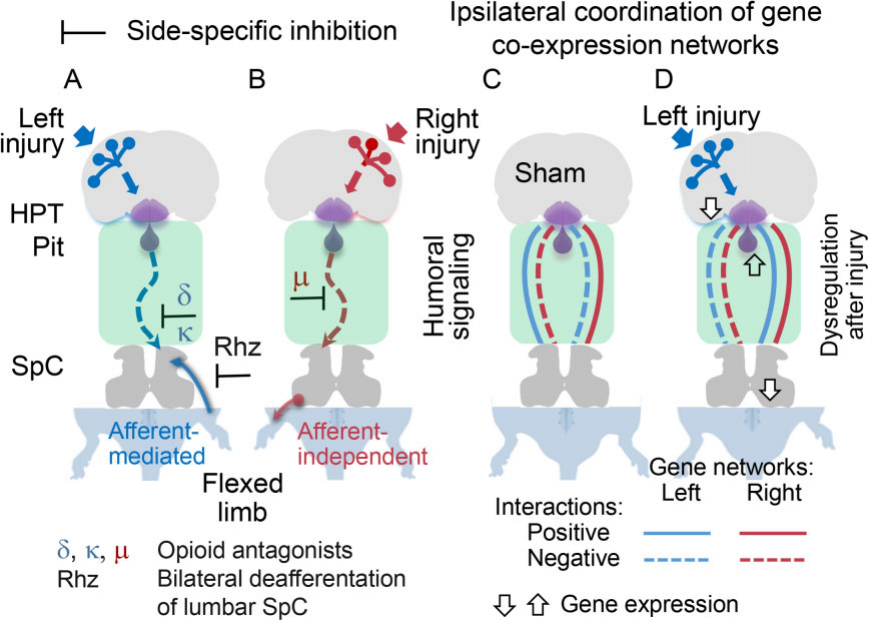
A model of the bipartite asymmetric T-NES that enables the left-right side-specific signaling from the brain to the lumbar spinal cord (SpC) through the humoral pathway. The left (A) and right (B) T-NES counterparts mediate the contralateral effects of the left-side and right-side brain injury. A unilateral brain lesion stimulates the release of side-specific neurohormones from the hypothalamus (HPT) and pituitary gland (Pit) into the blood, they bind to neuronal receptors that are lateralized in the spinal cord^13^,^63^ or peripheral neuronal endings, and induce contralateral responses, e.g., hindlimb flexion. The δ- and κ-opioid receptors may control signaling from the brain injured on the left side (left UBI), whereas the µ-opioid receptors may control signaling after the right UBI. The endogenous opioid peptides could differentially convey signals from the left and right hemispheres through the humoral pathway or control their processing in the hypothalamus or spinal cord. Although the left and right T-NES counterparts are not mirror symmetric to each other in their neural mechanisms, they produce overall symmetric functional responses, such as flexion of the right and left hindlimbs, respectively. The effects of the left T-NES but not right T-NES may require an afferent input and depend on spinal reflexes. The right T-NES effects may develop through activation of motoneurons or changes in the neuromuscular system. (C and D) The T-NES-mediated ipsilateral crosstalk between the hypothalamic and lumbar spinal cord gene expression networks and its reorganization in response to a unilateral brain lesion. Ipsilateral interactions between the hypothalamus and spinal cord are depicted in each network as positive if the proportion of positive correlations is >0.5 and negative if it is <0.5. Arrows show the direction of changes in gene expression levels induced by the left UBI. The ipsilateral correlations significantly differ between the dominant left gene expression network and dominant right gene expression network on each body side and for both networks between the sides. Only interactions of the left networks were significantly perturbed by the left UBI. The patterns of interactions were similar or almost mirror-symmetric (allo-symmetric) for the left networks on the left side and the right networks on the right side and for the right networks on the left side and the left networks on the right side. The diagonal (contralateral) inter-regional interactions were not significant.

The UBI-induced signaling is binary, either left- or right-sided. This could determine the bipartite structure of the T-NES that by encoding and decoding hormonal messages, may selectively propagate the effects of either left or right brain lesion (Figure 7A and B). The bipartite structure is supported by the findings that the left and right side-specific T-NES functions are differentially affected by selective opioid antagonists. The δ-antagonist NTI and the κ-antagonist BNI both inhibited HL-PA after left, but not after right UBI, whereas the µ-antagonist FNA interfered with the effects of right but not left UBI (Figure 7A and B). Of note, the opioid antagonists differentially blocked the effects of left and right UBI in rats with intact spinal cords.^13^ The signaling from the injured brain that causes the asymmetric hindlimb response in that study may be mediated by both neural and endocrine mechanisms. The preferred side of inhibition by δ- and µ-antagonists was the same in rats with intact^13^ and completely transected (this study) spinal cords. However, the side affected by the κ-antagonist was different between these groups of animals. Thus, opioid mechanisms through different receptor subtypes may allow flexibility of outcome based on synergy or antagonism of neural and endocrine pathways.

Translation of the T-NES humoral messages into the left-right side-specific hindlimb responses may occur in lumbar neural circuits or at peripheral nerve endings. We previously demonstrated that opioid peptides, synthetic opioids, and Arg-vasopressin may induce HL-PA after their intravenous or intrathecal administration into rats with intact brain but with completely transected spinal cords.^12^,^14^,^70–73^ The striking finding was that the side of the flexed limb depended on the compound administered. The µ/δ-opioid agonist Met-enkephalin, and the κ-opioid agonists dynorphin, bremazocine, and U-50,488 all induced flexion of the left hindlimb. In contrast, the δ-agonist Leu-enkephalin, β-endorphin, and Arg-vasopressin cause the right limb to flex.^12^,^71^,^72^,^74^ After brain injury, these neurohormones may be released from the endocrine glands into the bloodstream and induce side-specific effects through their receptors lateralized in the spinal cord or the peripheral afferent and motor nerve terminals. In the cervical and lumbar spinal cord, the expression of the opioid receptors is lateralized to the left, and the proportions of their subtypes and their co-expression patterns differ between the left and right sides.^13^,^63^ Activation of lateralized receptors by endogenous neurohormones may represent a decoding mechanism in the bipartite T-NES.

Bilateral deafferentation of the lumbar segments in animals with transected cervical spinal cords did not interfere with HL-PA caused by the right UBI, suggesting that spinal reflexes are not involved, while the asymmetry may persist due to activation of motoneurons or changes in neuromuscular system. In contrast, bilateral lumbar rhizotomy abolished HL-PA formed after the left UBI, suggesting that its maintenance requires an afferent input and depends on spinal reflexes. Thus, the left and right T-NES counterparts may act through different neural mechanisms and enable overall mirror-symmetric outcomes that is flexion of either right and left hindlimb, respectively (Figure 7A and B).

The asymmetric spinal processing of the effects of UBI is in agreement with other findings regarding spinal cord asymmetries.^11^,^63^,^75–81^ Three-quarters of cervical spinal cords are asymmetric with a larger right side.^80^ Mono- and polysynaptic spinal reflexes showed rightward lateralization.^11^,^77–79^ Lateralized signals from an injured brain that target spinal circuits may be clinically relevant. Patients with stroke and cerebral palsy often do not relax their muscles—they are tonically constricted without any voluntary command. This phenomenon is defined as “stretch- and effort-unrelated sustained involuntary muscle activity following central motor lesions” and is called spastic dystonia.^82–84^ This form of muscle overactivity may have a central mechanism^83^,^85^ that does not depend on afferent input in contrast to spasticity based on exacerbated reflex excitability.^86^ In this respect, HL-PA developed in rats after the right-side injury may be mechanistically similar with spastic dystonia and could model this human neuropathology. These animal findings suggest that spastic dystonia may affect more frequently the left than right lower limb in clinical cases.

### Lateralized Crosstalk between the Hypothalamus and Spinal Cord

We previously reported that gene expression patterns in the spinal cord are lateralized and affected by left UBI through a humoral pathway with clear differences between the contra- and ipsilesional sides.^11^,^12^,^63^ Here, we showed that expression of the neurohormonal and neuroplasticity-related genes was also different between the left and right sides in the hypothalamus and affected by a unilateral cortical lesion in this area. Left UBI decreased the expression levels of a subset of these genes in the ipsilesional hypothalamus with no significant changes on the right side. In the pituitary gland, left UBI resulted in elevation of expression of *Avpr1b* that encodes the Arg-vasopressin V1B receptor. Arg-vasopressin may induce HL-PA with flexion of the right limb in rats with intact brain and mediate the effects of left UBI on the hindlimb posture.^12^ These effects were blocked by SSR-149415, the selective antagonist of the V1B receptor that is mainly expressed in the anterior pituitary.^87^ It was hypothesized that Arg-vasopressin acting through the V1B receptor on the pituitary corticotropes stimulates the release of the proopiomelanocortin-derived β-endorphin that produces HL-PA with the right hindlimb flexion.^12^ Changes in V1B receptor expression suggest plasticity in the Arg-vasopressin system that signals from the injured brain.

#### The Left-Right Side-Specific Gene Co-expression Networks

Analysis of gene–gene co-expression patterns suggests that the neurohormonal, neuropeptide, and neuroplasticity-related genes form the left and right gene co-expression networks in the hypothalamus and spinal cord. It also suggests that these networks are laterally coordinated across these 2 regions and that UBI perturbs this coordination. In both regions, pairwise gene–gene correlations internal to the LdN and RdN were generally positive, while those between the networks were mostly negative, suggesting an antagonistic relation between the 2 networks. The hypothalamus–spinal cord correlation patterns were strikingly different between the LdN and RdN. In the proportion of positive correlations, the ipsilateral hypothalamus–spinal cord co-expression pattern for the LdN genes displayed marked left–right asymmetry (Figure 7C). Similarly, the pattern of ipsilateral inter-area correlations for the RdN was also asymmetric, but the direction was opposite to that of LdN. At the same time, the patterns were almost mirror-symmetric between the LdN on the left side and the RdN on the right side, and between the RdN on the left side and the LdN on the right side (Figure 7C). These nearly perfect in their structure ensembles can be defined as “allo-symmetric.” In contrast to the ipsilateral patterns, the diagonal (contralateral) inter-area correlations were similar between LdNs and RdNs and remained unaffected by UBI. Asymmetry of both LdN and RdN and “allo-symmetry” between them were characteristics of the control group and were impaired by the unilateral brain lesion. For example, the direction of the left-right LdN asymmetry was reversed after the left UBI.

#### Functional Implications

Formation of the LdN and RdN by neurohormonal, neuropeptide, and neuroplasticity-related genes, along with the asymmetry and “allo-symmetry” of their patterns were revealed in animals with completely transected spinal cords, suggesting that they were established by the T-NES and remodeled after TBI through the humoral pathway (Figure 7C and D). The functional role of the LdN and RdN may be to amplify the inherently weak lateralized effects of individual neurohormones and to strengthen the left and right side-specific regulations by these molecules. This mechanism could operate within and between the left and right halves of CNS areas, and across CNS regions and their left and right sides along the neuraxis.

The LdN *Avp* and *Pomc* genes give rise to Arg-vasopressin and β-endorphin that induce right hindlimb flexion in animals with intact brain and may mediate the effects of the left-side UBI.^12^,^74^ In contrast, Met-enkephalin and dynorphin derived from *Penk* and *Pdyn*, constituents of the RdN, produce flexion of the left hindlimb.^14^,^70–72^ The hypothalamic neurohormones oxytocin and TRH, whose genes are constituents of the LdN, and gonadotropin-releasing hormone, transcribed from the RdN *Ghrh1* gene, produce lateralized responses and may regulate lateralized brain functions. The oxytocin receptors mediate the effects of this peptide released from the hypothalamus on pup retrieval behavior through activation of the auditory cortex on the left but not right side.^88^ In the human brain, the opioid, Arg-vasopressin, and oxytocin systems are lateralized and could mediate lateralized responses.^89–92^ TRH showed a substantial left side predominance in the hypothalamic nuclei and produced an asymmetric behavior.^93–95^ Gonadotropin-releasing hormone is asymmetrically expressed in the hypothalamus and involved in asymmetric circadian regulation of reproductive functions.^96–99^

An intriguing possibility is that neurohormones and neuropeptides with asymmetric actions may be organized into the left- and right-sided functional networks that control the entire left and right hemispheres, respectively. These networks may differ between the hemispheres, whereas their integral activities may be balanced between the left and right sides. This hypothesis was addressed by analysis of the effects of peptide pools prepared from the left and right brain hemispheres of intact rats using the HL-PA model.^100–103^ The binary, the left-sided, or right-sided responses to peptide administration were assessed in rats with intact brain but completely transected spinal cords. Intrathecal administration of the extracts resulted in development of HL-PA. Remarkably, the direction of the asymmetry depended on whether an extract was prepared from the left or right hemisphere. The “left” extract induced flexion of left hindlimb, while the right hindlimb was flexed after administration of peptides from the right hemisphere. No asymmetry was formed after administration of the total peptide pool prepared from the whole brain. Thus, peptides with side-specific actions were lateralized in the brain, and the integral activity of the “left” and “right” peptide factors was balanced between the hemispheres. Biochemical analysis demonstrated that factors inducing HL-PA were multiple short peptides that remain to be identified.^103^

### Limitations

T-NES was identified in acute experiments lasting 3–6 h after UBI in anesthetized male rats with completely transected spinal cords. Hence, a role for this phenomenon in the persistent biological and pathophysiological processes requires further investigation. This can be addressed by analyzing the protracted effects of unilateral TBI or stroke on contralateral postural and motor deficits in subchronic experiments with awake animals whose spinal cords are completely transected to disable neural pathways. The methodology may consist of a combined behavioral, electrophysiological, and biomechanical assessment of hindlimb function while performing body-weight-supported stepping. Additionally, it is necessary to evaluate the efficacy of T-NES in female animals, considering their different endocrine status.

HL-PA, a proxy for neurological deficits, enabled the discovery and characterization of T-NES. The model is binary, featuring 2 qualitatively distinct responses that are generated on either the left- or right-side. HL-PA can model human neurological deficits, such as hemiparesis, hemiplegia, and spastic dystonia secondary to TBI and stroke. It allows for reliable and quick testing of multiple hypotheses and is relatively easy to perform. On the other hand, HL-PA cannot be analyzed in awake animals, and knowledge of its mechanisms is limited. The neural pathways that may mediate signals from the injured brain region to the hypothalamic–pituitary system, the coding and decoding molecular mechanisms, and “left” and “right” hormones released by this system, as well as the afferent and central HL-PA mechanisms, still require exploration.

In the molecular part of this study, the categorization of genes into LdN and RdN was based on the direction of their lateralization, but not on statistical significance or range of asymmetry. The selection of neuroplasticity-related genes was arbitrary, and the selected set was not comprehensive. Nevertheless, robust differences between the LdN and the RdN in their intra- and inter-area correlation patterns were uncovered, suggesting that a substantial part of these genes was correctly assigned to a respective network. The analyzed set of neurohormonal and neuroplasticity-related genes, although relatively small, allowed us to identify the LdN and RdN. Further transcriptome-wide analysis could reveal a complete structure of the left-right-specific gene expression networks.

## Conclusions

In addition to descending neural tracts, the contralateral effects of brain injury may also be mediated by the endocrine system through the humoral pathway.^12^ A third potential signaling pathway through the paravertebral sympathetic chain of ganglia has been ruled out in the present study by the results obtained from rats with transected cervical spinal cords. Here, we uncovered the organizational principle of T-NES; in particular, its bipartite structure and functional and molecular asymmetries. The left and right T-NES counterparts mediate the effects of left and right brain injury, respectively, and enable overall mirror-symmetric functional responses (eg, right and left hindlimb flexion). However, the neural and neurohormonal mechanisms underlying these responses are different. The maintenance of the left T-NES effects, but not those of the right T-NES required afferent input. Activation of motoneurons but not hindlimb reflexes may underlie the effects of the right UBI. Antagonists of δ-, κ-, and µ-opioid receptors differentially inhibited neurohormonal signaling from the left and right hemispheres. Thus, endogenous opioid peptides may convey the “left” and “right” T-NES signals via the humoral pathway or differentially control their processing in the hypothalamus or spinal cord.

Analysis of gene–gene co-expression patterns revealed left-right side-specific gene co-expression networks and their ipsilateral coordination across the hypothalamus and spinal cord. The ipsilateral interactions differed between the left and right gene networks on each side of the body and for both networks between the sides of the body. Left UBI perturbed these patterns by affecting the LdN. The findings suggest the side-specific ipsilateral endocrine crosstalk between the hypothalamus and lumbar spinal cord that coordinates molecular processes between these regions and is reorganized in the response to a unilateral brain lesion.

Functional specialization of the left and right hemispheres is an organizing principle of the brain.^104–107^ Lateralized processes may be regulated by the side-specific neurohormonal mechanisms that operate on either the left or right side.^6^,^14^,^63^,^76^,^88^,^89^,^91^,^108–113^ Our findings suggest a more general role for the lateralized neuroendocrine system. A fundamental feature of the bilaterian body is its symmetrical organization and function, which requires robust control of the balance between left- and right-sided processes. We hypothesize that the bipartite T-NES is part of this left-right-specific control mechanism. T-NES may be based on the lateralized neurohormonal networks and may act locally (eg, within brain and spinal cord areas) or along the neuraxis by signaling from the left and right hemispheres to the ipsilateral or contralateral side of the body. A unilateral brain lesion could shift this balance to the left or right, depending on the side of injury, and thereby disrupt left-right side-specific neurohormonal control, leading to asymmetric functional impairments. From a clinical point of view, it is essential to evaluate the contribution of neural and endocrine pathways to protracted neurological deficits after TBI and stroke, including hemiparesis and hemiplegia, and to develop pharmacological means to restore the impaired neurohormonal balance.

## Glossary

**Asymmetry index**

AI, the asymmetry index.

AI_L/R_, the left / right asymmetry index computed as log_2 _(L/R), where L and R are values for the left and right sides. The AI_L/R_ was analyzed both for the resistance to stretch (*W*) and for the gene expression levels.

AI_C/I_, the contralesional/ipsilesional asymmetry index computed as log_2 _(C/I), where C and I are values for the contralesional and ipsilesional sides. It was used in the analysis of *W*.

**Brain injury**

UBI, a unilateral brain injury.

L-UBI, a left unilateral brain injury.

R-UBI, a right unilateral brain injury.

**CNS areas**

HPT, the hypothalamus.

Pit, the pituitary gland.

SpC, the spinal cord.

**Correlation analysis**

Coordination strength, magnitude of correlations (absolute value of the correlation coefficients) averaged across pairwise correlations.

**Hindlimb postural asymmetry analysis**

HL-PA, hindlimb postural asymmetry.

PAS, the postural asymmetry size in mm, with the direction of the asymmetry shown as negative values for left hindlimb flexion and positive values for right hindlimb flexion.

MPA, the magnitude of postural asymmetry size in mm.

*P*_A_, the probability to develop HL-PA with the MPA >1 mm.

**Musculo-articular resistance to stretching**

*W*, the work in gm × mm for passive hindlimb musculo-articular resistance to stretching.

*W*_L-R_, the difference in the work applied to stretch the left (L) and right (R) hindlimbs.

*W*_L/R_, the left/right asymmetry index computed as log_2 _(*W*_L_/*W*_R_), where *W*_L_ and *W*_R_ are *W* values for the left and right hindlimbs.

*W*_C-I_, the difference in the work applied to stretch the contralesional (C) and ipsilesional (I) hindlimbs.

**Molecular analysis**

LdN, the left dominant gene co-expression network consisting of genes with the asymmetry index AI_L/R _> 0.

RdN, the right dominant gene co-expression network consisting of genes with the asymmetry index AI _L/R _< 0.

lm, the left module constituted by gene transcripts of the left half of the hypothalamus or the spinal cord.

rm, the right module constituted by gene transcripts of the right half of the hypothalamus or the spinal cord. 

**Selective opioid antagonists**

BNI, κ-opioid antagonist nor-binaltorphimine binaltorphimine.

FNA, µ-opioid antagonist β-funaltrexaminefunaltrexamine.

NTI, δ-opioid antagonist naltrindole.

**Statistical terms**

95% HPD, the highest posterior density credible interval, within which an unobserved parameter value falls with 95% probability. This is a Bayesian analog of 95% CI.

Significant asymmetry, 95% HPD does not include zero and adjusted *P*-value < .05.

Contrast, the median of one group minus the median of another group. Denoted as ∆.

Contrast of contrasts, difference between 2 contrasts. Denoted as ∆∆.

FC, fold change.

## Supplementary Material

zqae013_Supplemental_File

## Data Availability

Data supporting the findings of this study and all codes used for analysis are available within the article, its Supporting Information and on https://github.com/YaromirKo/biostatistics-nms.
