# Supplementary material for: The Left-Right Side-Specific Neuroendocrine Signaling from Injured Brain: An Organizational Principle
Source: Function (Oxf). 2024 Mar 14;5(4):zqae013. doi: 10.1093/function/zqae013 (PMC11237900; doi:10.1093/function/zqae013)
Supplement: zqae013_Supplemental_File [file zqae013_supplemental_file.docx]

**Supplementary data**

The left-right side-specific neuroendocrine signaling from injured brain: an organizational principle

*Running Title*: Left-right sided endocrine signaling

 Hiroyuki Watanabe^1,2,*^, Yaromir Kobikov^3,*^, Olga Nosova^1,*^, Daniil Sarkisyan^1,4,*^, Vladimir Galatenko^5^, Liliana Carvalho^6^, Gisela H. Maia^7,8,9^, Nikolay Lukoyanov^6,9,10^, Igor Lavrov^11^, Michael H. Ossipov^12^, Mathias Hallberg^1^, Jens Schouenborg^13^, Mengliang Zhang^2,13,‡^ and Georgy Bakalkin^1,‡^

^1^ Department of Pharmaceutical Biosciences, Uppsala University, Uppsala, Sweden

^2^ Department of Molecular Medicine, University of Southern Denmark, Odense, Denmark

^3^ Volunteer Associate Department of Pharmaceutical Biosciences, Uppsala University. Present address: Tashkent, Uzbekistan

^4^ Department of Immunology, Genetics and Pathology and Science for Life Laboratory, Uppsala University, Uppsala, Sweden

^5^ Evotec International GmbH, Göttingen, Germany

^6^ Departamento de Biomedicina da Faculdade de Medicina da Universidade do Porto, Portugal

^7^ Centro de Investigação em Saúde Translacional e Biotecnologia Médica (TBIO)/Rede de Investigação em Saúde (RISE-Health), Escola Superior de Saúde, Instituto Politécnico do Porto, Portugal

^8^ Medibrain, Vila do Conde, Portugal

^9^ Brain Research Institute, Porto, Portugal

^10^ i3S - Instituto de Investigação e Inovação em Saúde, Universidade do Porto, Portugal

^11^ Department of Neurology, Mayo Clinic, Rochester, MN, USA,

^12^ Department of Pharmacology, University of Arizona College of Medicine, Tucson, AZ, USA

^13^ Neuronano Research Center, Department of Experimental Medical Science, Lund University, Lund, Sweden

^*^ Contributed equally to this work

‡ Co-senior authors

**Corresponding author:**

Georgy Bakalkin: Georgy.Bakalkin@uu.se


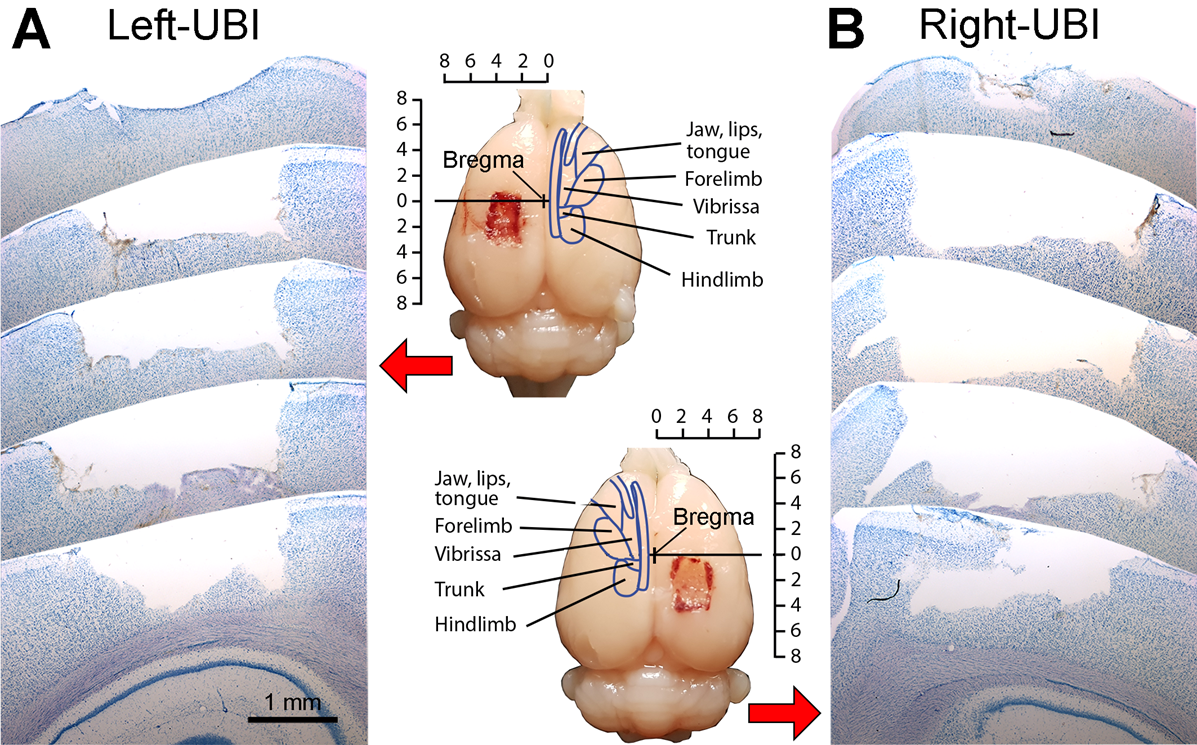


**Figure 1—figure supplement 1. Lesion area in the hindlimb sensorimotor cortex from rats with the left and right unilateral brain injury (UBI) rat.** (**A,B**) Five consequent toluidine blue‐stained cortical sections at an equal distance (1000 μm) across the lesion site in the left and right hemispheres 3 hours after the UBI. Macrographs in the middle show the same brains before sectioning. The delineations on the cortex represents somatotopically organized primary motor cortex (modified from Hall, R. D., & Lindholm, E. Organization of motor and somatosensory neocortex in the albino rat. Brain Res, 1974, 66, 23-38). The coordinates are in mm. The scale bar in (**A**) is also valid for (**B**).


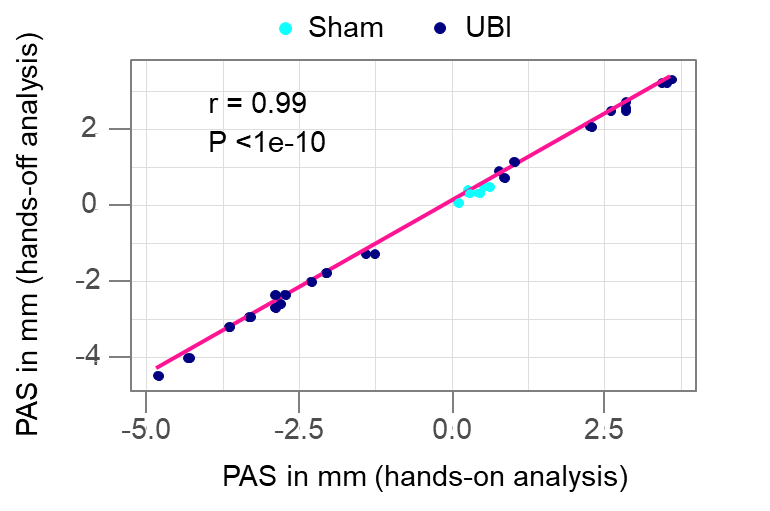


**Figure 1—figure supplement 2. Pearson correlation between the postural asymmetry size (PAS) analyzed by the hands-off and hands-on assay.** Data were combined for left UBI, right UBI and sham surgery groups of rats with transected cervical spinal cords, which were analyzed 3 hours after brain surgery.

**Source data:** The EXCEL source data file “masterfile-210807.xlsx ”.

**
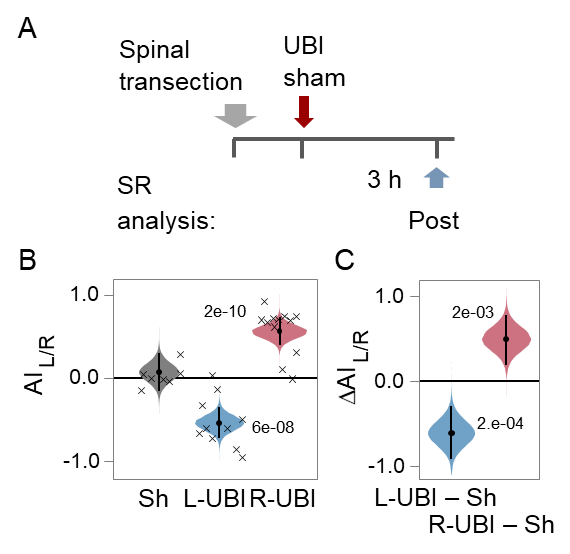
**

**Figure 1—figure supplement 3. Asymmetry in the hindlimb stretching resistance (SR) in rats with completely transected cervical spinal cord: effects of UBI.** (**A**) Experimental design. Spinal cord transection was followed by left UBI (L-UBI), right UBI (R-UBI) or sham surgery (Sh). (**B**,**C**) Stretching force analyzed three hours after UBI or sham surgery (Post; L-UBI, n = 10; R-UBI, n = 12; and sham surgery, n = 7). The UBI effects were analyzed as changes in the asymmetry index for left and right hindlimbs AI_L/R_ = log_2_ (W_L_/ W_R_). (**C**) Differences (contrast) between the UBI and sham surgery groups. The AI_L/R_ and contrasts are plotted as median (black circles), 95% HPD, and posterior density (colored distribution) from Bayesian regression. Crosses denote the AI_L/R_ values for individual rats. Asymmetry and contrasts among the groups were deemed significant, with a 95% HPD not encompassing zero and adjusted P values of ≤ 0.05. Adjusted P values are presented numerically on the plots.

**Source data:** The EXCEL source data file “masterfile-210807.xlsx” and source data folder “/HL-PA/data/SF/”.

**
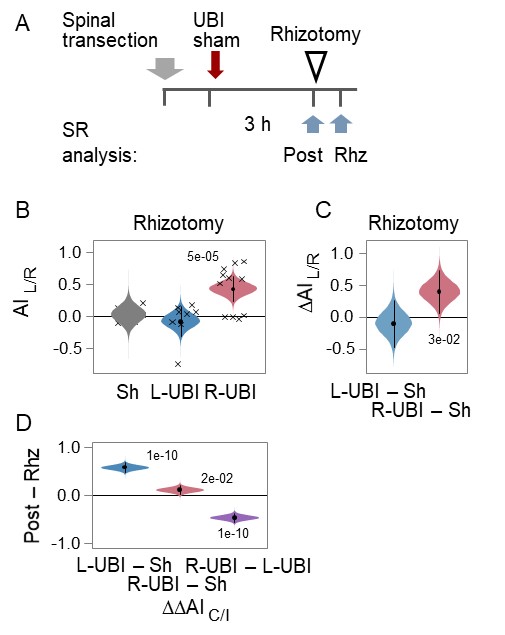
**

**Figure 2—figure supplement 1. Asymmetry in the UBI-induced hindlimb stretching resistance (SR) in rats with completely transected cervical spinal cord: effects of bilateral deafferentation of the lumbar spinal cord.** (**A**) Experimental design. Spinal cord transection was followed by left UBI (L-UBI), right UBI (R-UBI) or sham surgery (Sh). Stretching force was analyzed three hours after UBI or sham surgery (Post; L-UBI, n = 10; R-UBI, n = 12; and sham surgery, n = 7); and after bilateral rhizotomy (Rhz) in the subset of rats (L-UBI, n = 8; R-UBI, n = 11; and sham surgery, n = 7). The UBI effects were analyzed as changes in the asymmetry index for left and right hindlimbs AI_L/R_ = log_2_ (W_L_ / W_R_) (**B,C**), and for contra- and ipsilesional hindlimbs AI_C/I_ = log_2_ (W_C_ / W_I_) (**D**). (**B**,**C**) The AI_L/R_ and contrasts between the UBI and sham surgery groups after rhizotomy. (**D**) The effects of rhizotomy on differences in AI_C/I_ between L-UBI, R-UBI and sham surgery were analyzed as contrast of contrasts i) between L-UBI and sham surgery: ΔΔAI_C/I_ = [(L-UBI _Post_ – Sh _Post_) – (L-UBI _Rhz_ – Sh _Rhz_)]; ii) between R-UBI and sham surgery: ΔΔAI_C/I_ = [(R-UBI _Post_ – Sh _Post_) – (R-UBI _Rhz_ – Sh _Rhz_)]; and iii) between R-UBI and L-UBI sham surgery: ΔΔAI_C/I_ = [(R-UBI _Post_ – L-UBI _Post_) – (R-UBI _Rhz_ – L-UBI _Rhz_)].

Crosses denote the AI_L/R_ values for individual rats. The AI_L/R_, contrast and contrast of contrasts are plotted as median (black circles), 95% HPD (black lines), and posterior density (colored distribution) from Bayesian regression. Significant effects between the groups: 95% HPD did not include zero, and adjusted P-values were ≤ 0.05. Adjusted P values are presented numerically on the plots.

**Source data:** The EXCEL source data file “masterfile-210807.xlsx” and source data folder “/HL-PA/data/SF/”.

**Figure 3—figure supplement 1**

**Table 1. Animal Group Design and Number of Rats Analyzed in Opioid Antagonist Studies.** Rats with completely transected cervical spinal cord and left UBI (L-UBI) or right UBI (R-UBI) were treated with nor-binaltorphimine (BNI), β-funaltrexamine (FNA), naltrindole (NTI) or naloxone, or were used as controls (Ctrl). The Ctrl1 was a control group for BNI and FNA, and the Ctrl2 was a control group for NTI and naloxone. Both control groups consisted of UBI rats that were not treated with the antagonists. Since were no statistically significant differences in both MPA and W_C-I_ between the left UBI and right UBI subgroups in either control group, and the two subgroups were combined in each Ctrl1 and Ctrl2 group for statistical analysis. In design B (**Figure 3**), the NTI- and Nal-treated groups (Ant2) and the control group (Ctrl) were composed of rats with MPA exceeding 1.5 mm 3 h after UBI. Ctrl2 rats were treated with saline 3 h after UBI or not treated. No significant differences were found between these subgroups in either MPA or W_C-I_, allowing them to be combined into one group (Ctrl2).

| Rat group / Treatment | Left UBI rats | Right UBI rats |
| --- | --- | --- |
| Ctrl1 | 17 | 13 |
| BNI | 7 | 9 |
| FNA | 8 | 8 |
| Ctrl2 untreated with saline | 12 | 12 |
| Ctrl2 treated with saline | 4 | 3 |
| NTI | 8 | 8 |
| Naloxone | 7 | 7 |

**Figure 4—figure supplement 1**

**Table 2.** Genes of hypothalamic releasing and inhibitory hormones and neuropeptide neurotensin, and PCR probes for analysis of their expression levels (Bio-Rad Laboratories, CA, USA).

| Gene name | Gene symbol | Assay ID | Channel |
| --- | --- | --- | --- |
| *Corticotropin releasing hormone* | *Crh* | qRnoCEP0026204 | FAM |
| *Growth hormone releasing hormone* | *Ghrh* | qRnoCIP0029554 | HEX |
| *Gonadotropin releasing hormone 1* | *Gnrh1* | qRnoCIP0025960 | FAM |
| *Neurotensin* | *Nts* | qRnoCIP0026101 | FAM |
| *Somatostatin* | *Sst* | qRnoCEP0031118 | FAM |
| *Thyrotropin Releasing Hormone* | *Trh* | qRnoCEP0026268 | HEX |

**Figure 4—figure supplement 2**

**Table 3.** Genes of the endogenous opioid system, and PCR probes for analysis of their expression levels (Bio-Rad Laboratories, CA, USA).

| Gene name | Gene symbol | Assay ID | Channel |
| --- | --- | --- | --- |
| *Opioid receptor delta 1* | *Oprd1* | qRnoCEP0029668 | FAM |
| *Opioid receptor kappa 1* | *Oprk1* | qRnoCIP0029310 | HEX |
| *Opioid receptor mu 1* | *Oprm1* | qRnoCEP0024902 | FAM |
| *Prodynorphin* | *Pdyn* | qRnoCEP0025357 | FAM |
| *Proenkephalin* | *Penk* | qRnoCEP0029455 | HEX |
| *Proopiomelanocortin* | *Pomc* | qRnoCIP0024350 | HEX |

**Figure 4—figure supplement 3**

**Table 4.** Genes of the oxytocin-vasopressin systems, and PCR probes for analysis of their expression levels (Bio-Rad Laboratories, CA, USA).

| Gene name | Gene symbol | Structure/ structures analyzed* | Assay ID | Channel |
| --- | --- | --- | --- | --- |
| *Arginine vasopressin* | *Avp* | HPT, PG | qRnoCEP0023611 | FAM |
| *Arginine vasopressin receptor 1A* | *Avpr1a* | PG, SpC | qRnoCEP0023750 | FAM |
| *Arginine vasopressin receptor 1B* | *Avpr1b* | PG | qRnoCEP0037165 | FAM |
| *Arginine vasopressin receptor 2* | *Avpr2* | PG | qRnoCEP0030097 | FAM |
| \| *Oxytocin* \| *Oxt* \| \| --- \| --- \| | *Oxt* | HPT, PG | qRnoCEP0031434 | HEX |

*Structures analyzed: HPT – hypothalamus, PG – pituitary gland, SpC – spinal cord.

**Figure 4—figure supplement 4**

**Table 5.** Neuroplasticity-related genes, and PCR probes for analysis of their expression levels (Bio-Rad Laboratories, CA, USA).

| Gene name | Gene symbol | Assay ID | Channel |
| --- | --- | --- | --- |
| *Activity-regulated cytoskeleton-associated protein* | *Arc* | qRnoCEP0027389 | HEX |
| *Brain-derived neurotrophic factor* | *Bdnf* | qRnoCEP0026843 | HEX |
| *Fos proto-oncogene* | *cFos* | qRnoCEP0024078 | HEX |
| *Discs large MAGUK scaffold protein 4* | *Dlg4* | qRnoCIP0026242 | FAM |
| *Early growth response 1* | *Egr1* | qRnoCEP0022872 | FAM |
| *Growth associated protein 43* | *Gap43* | qRnoCIP0027599 | FAM |
| *Glutamate ionotropic receptor AMPA type subunit 1* | *GluR1* | qRnoCIP0030725 | FAM |
| *Glutamate ionotropic receptor NMDA type subunit 2a* | *Grin2a* | qRnoCIP0025244 | HEX |
| *Glutamate ionotropic receptor NMDA type subunit 2b* | *Grin2b* | qRnoCIP0023973 | HEX |
| *Homer scaffold protein 1* | *Homer-1* | qRnoCEP0023985 | FAM |
| *Proprotein convertase subtilisin/kexin type 6* | *Pcsk6* | qRnoCIP0045340 | FAM |
| *NFKB inhibitor alpha* | *Nfkbia* | qRnoCEP0026759 | HEX |
| *Synaptotagmin 4* | *Syt4* | qRnoCIP0029728 | FAM |
| *Transforming growth factor beta 1* | *Tgfb1* | qRnoCIP0031022 | HEX |

**Figure 4—figure supplement 5.** **The UBI effects on gene expression in the left and right hypothalamus**. Samples were dissected 3 h after left sham surgery (n = 11) or left UBI (n = 12). The expression levels are presented in the log_2_ scale as boxplots with median and hinges representing the first and third quartiles, and whiskers extending from the hinge to the highest/lowest value that lies within the 1.5 interquartile range of the hinge. Unadjusted P-values computed using Mann–Whitney test are shown. Fold changes in the left hypothalamus: 1.76x for *Crh* gene, 1.59x for *Sst* gene, 1.25x for *Bdnf* gene, 1.22x for *Syt4* gene, 2.49x for *Pomc* gene, and 1.63x for *Ghrh* gene.

**Source data:** The EXCEL source data file “Hypoth_SO_UBI.xlsx”.

**Figure 4—figure supplement 6.** **Correlation of the UBI-induced fold changes in the levels of gene expression between the left and right hypothalamus (HPT).** Log-scaled fold changes in expression levels of individual genes (log_2_FC, where FC is ratio of median expression levels of UBI and sham groups) are shown. Pearson correlation coefficient: 0.79, P = 5.4×10^-7^; Spearman’s rank correlation coefficient: 0.48, P = 0.010. In a linear model (logFC_right_ ≈ *a* logFC_left_ + *b*), *a* = 0.64 (95% confidence interval [0.45, 0.83]) and *b* = –0.02 (95% confidence interval [-0.12, 0.08]).

**Source data:** The EXCEL source data file “Hypoth_SO_UBI.xlsx”.

**Figure 4—figure supplement 7**

**Table 6.** Genes coding for pituitary hormones and PCR probes for analysis of their expression levels (Bio-Rad Laboratories, CA, USA).

| Gene name | Gene symbol | Assay ID | Channel |
| --- | --- | --- | --- |
| *Follicle stimulating hormone, subunit beta* | *Fshb* | qRnoCEP0028026 | FAM |
| *Glycoprotein hormones, alpha polypeptide* | *Cga* | qRnoCIP0024751 | FAM |
| *Growth hormone 1* | *Gh1* | qRnoCEP0025504 | HEX |
| *Luteinizing hormone, subunit beta* | *Lhb* | qRnoCEP0025468 | FAM |
| *Prolactin* | *Prl* | qRnoCEP0029733 | HEX |
| [*Thyroid stimulating hormone, beta subunit*](https://www.ncbi.nlm.nih.gov/gene/22094) | *Tshb* | qRnoCEP0030177 | HEX |

**Figure 4—figure supplement 8.** **The UBI effects on the levels of gene expression in the pituitary gland.** mRNAs were analyzed in the pituitary isolated 3 h after left sham surgery (n = 11) or left UBI (n = 12). The expression levels presented in the log_2_ scale as boxplots with median and hinges representing the first and third quartiles, and whiskers extending from the hinge to the highest/lowest value that lies within the 1.5 interquartile range of the hinge. Unadjusted *P* computed using Mann–Whitney test is shown. FC = 1.17x for *Oxt* gene and FC = 1.63x for *Tshb* gene.

**Source data:** The EXCEL source data file “RD Hypophis_ Master file.xlsx”.


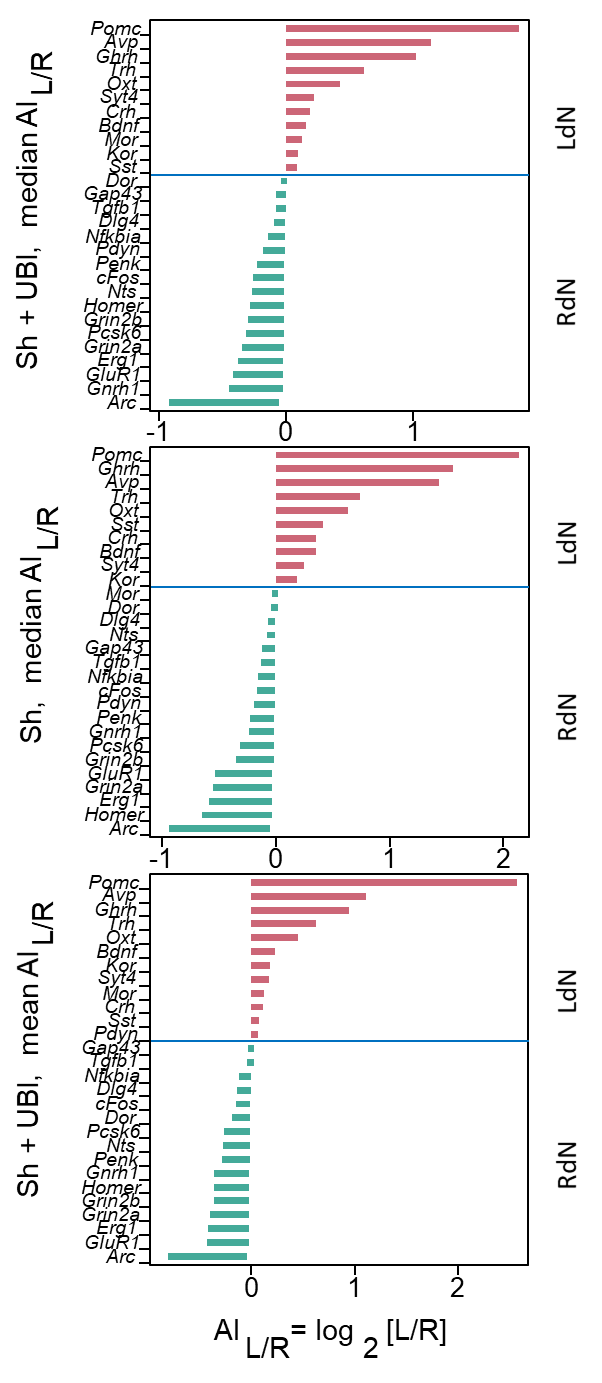


**Figure 4—figure supplement 9**. Categorization of genes in the hypothalamus into the left (LdN) and right (RdN) dominant gene regulatory networks based on the asymmetry index AI_L/R_ = [log_2_(L/R)], where L and R are gene expression levels in left and right hypothalamus, respectively. Genes of the LdN and RdN were defined as those that with AI_L/R_ > 0 and AI_L/R_ < 0, respectively. Three categorization variants of genes into the networks were based on the AI_L/R_ values computed as i) median AI_L/R_ in the combined sham surgery and UBI group (variant 1); ii) median AI_L/R_ in the sham surgery group only (variant 2); and iii) mean AI_L/R_ in the combined sham surgery and UBI group (variant 3). All genes showed stable patterns between the LdN and RdN in three categorization variants besides *Mor* that wobbled. All three variants were used for analysis of correlation patterns.

**Source data:** The EXCEL source data file “Hypoth_SO_UBI.xlsx; Table III-S6 23 05 10.xlsx; raw_groups.xlsx”.


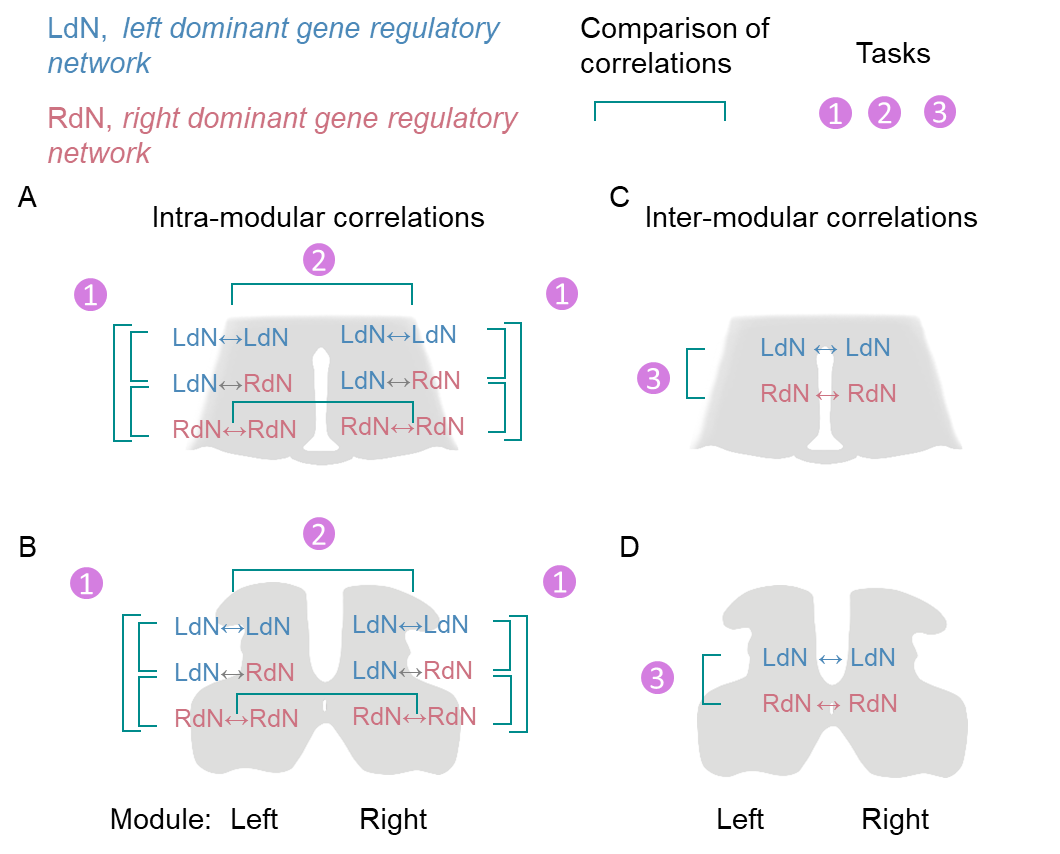


**Figure 4—figure supplement 10**. **Analysis of the LdN and RdN correlation patterns in the hypothalamus and spinal cord.** (**A,B**) Intra-modular correlation patterns and their comparisons depicted as Tasks 1 and 2. (**C,D**) Inter-modular correlation patterns and their comparisons depicted as Task 3. Correlations between two variables are shown by double head arrows.


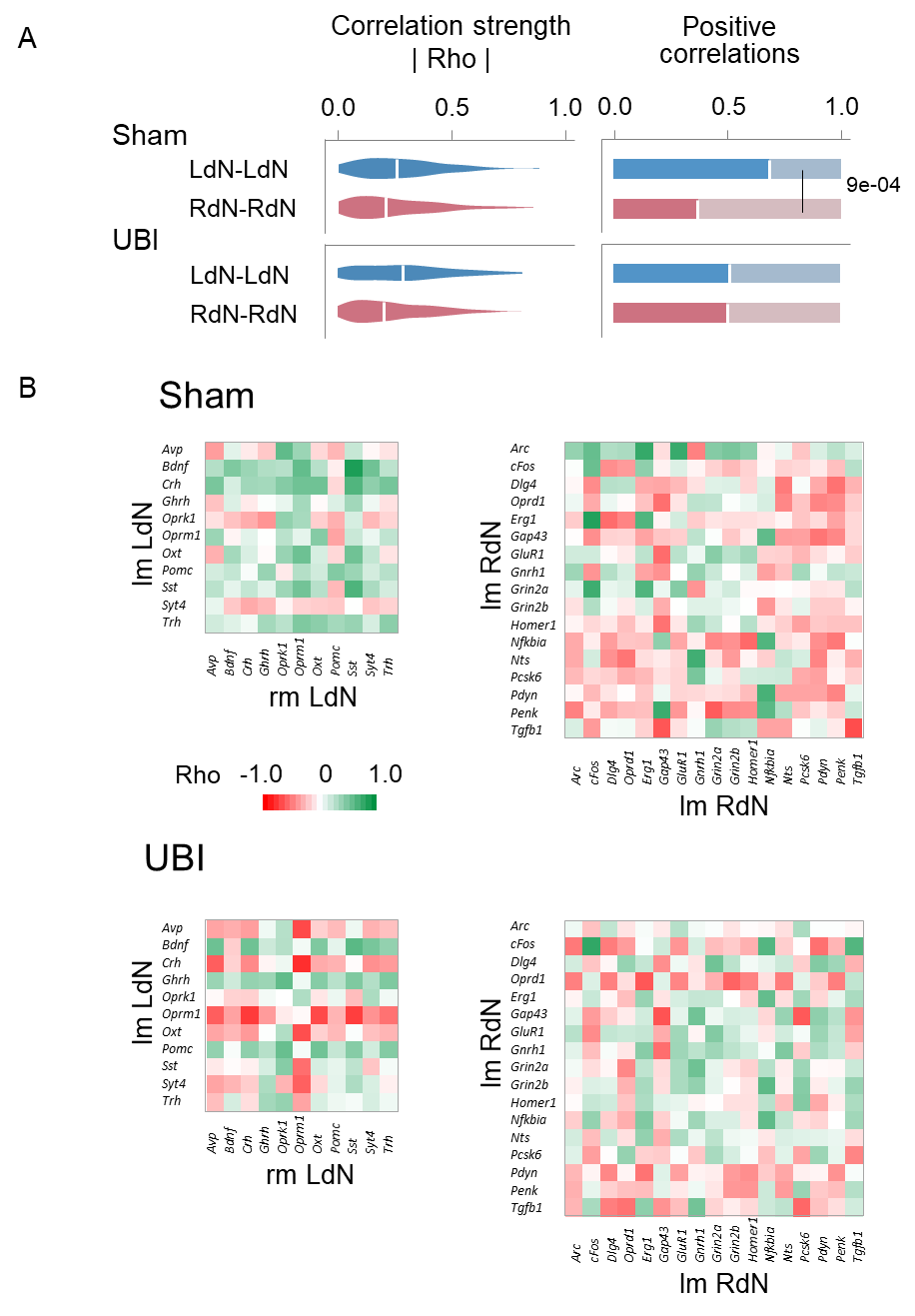


**Figure 4—figure supplement 11.** **Inter-modular correlations in the hypothalamus of the sham surgery and UBI rats.** Inter-modular correlations (lm – rm) are visualized in **Figure 4—figure supplement 10**. (**A**) Comparison of the coordination strength and the proportion of positive correlations between the inter-modular internal LdN correlations and the inter-modular internal RdN correlations, and for both networks between the sham surgery and UBI groups. P values were determined by permutation testing with Benjamini-Hochberg family-wise multiple test correction. Significance for contrasts was determined by analysis of three AI_L/R_ categorization variants (**Figure 4—figure supplement 9**); P value is shown for the categorization variant with the median AI_L/R_ of the combined sham surgery and UBI group. (**B**) Heatmaps for Spearman’s rank coefficients for pairwise gene-gene the inter-modular correlations of the LdN and RdN.

**Source data:** The EXCEL source data file “Hypoth_SO_UBI.xlsx; Table III-S6 23 05 10.xlsx; raw_groups.xlsx”.


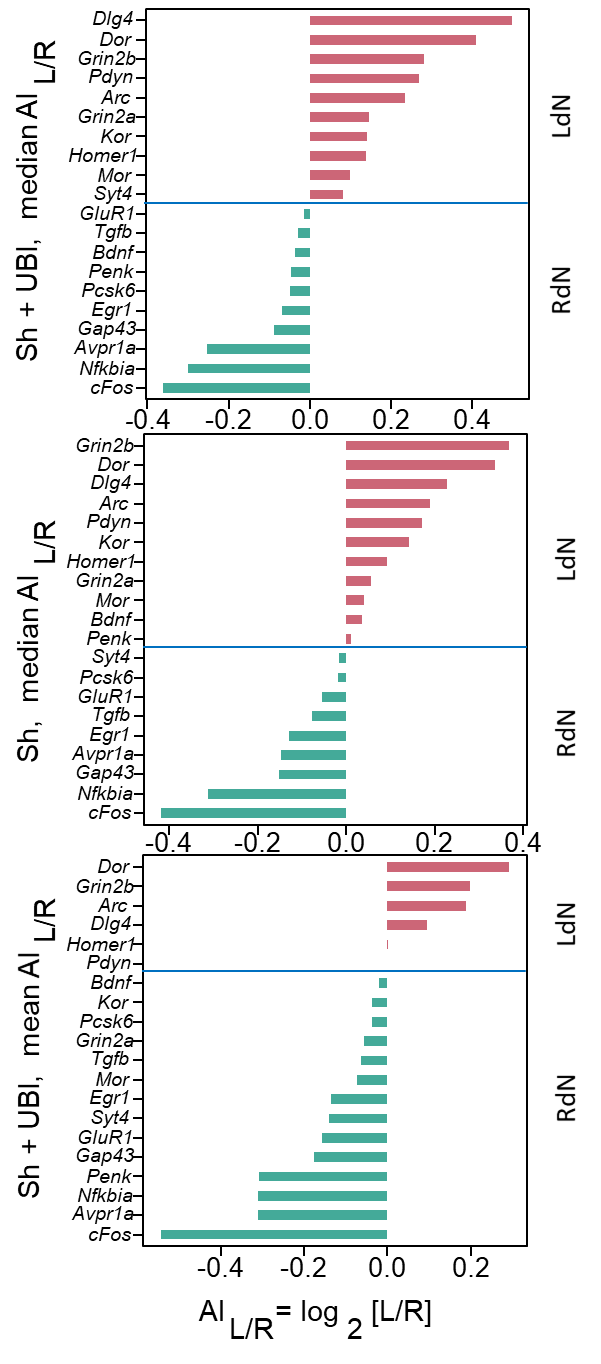


**Figure 5—figure supplement 1**. Categorization of genes in the spinal cord into the left (LdN) and right (RdN) dominant gene regulatory networks based on the asymmetry index AI_L/R_ = [log_2_(L/R)], where L and R are gene expression levels in left and right spinal cord, respectively. Genes of the LdN and RdN were defined as those that with AI_L/R_ > 0 and AI_L/R_ < 0, respectively. Three categorization variants of genes into the networks were based on the AI_L/R_ values computed as i) median AI_L/R_ in the combined sham surgery and UBI group (variant 1); ii) median AI_L/R_ in the sham surgery group only (variant 2); and iii) mean AI_L/R_ in the combined sham surgery and UBI group (variant 3). Five LdN genes and eight RdN genes showed stable patterns across the three variants, while seven genes with the AI_L/R_ values close to zero wobbled between the sides. All three variants were used for analysis of correlation patterns.

**Source data:** The EXCEL source data file “SpinalC_SO_UBI_Ctrl_RD_DD.xlsx; Table III-S6 23 05 10.xlsx; raw_groups.xlsx”.


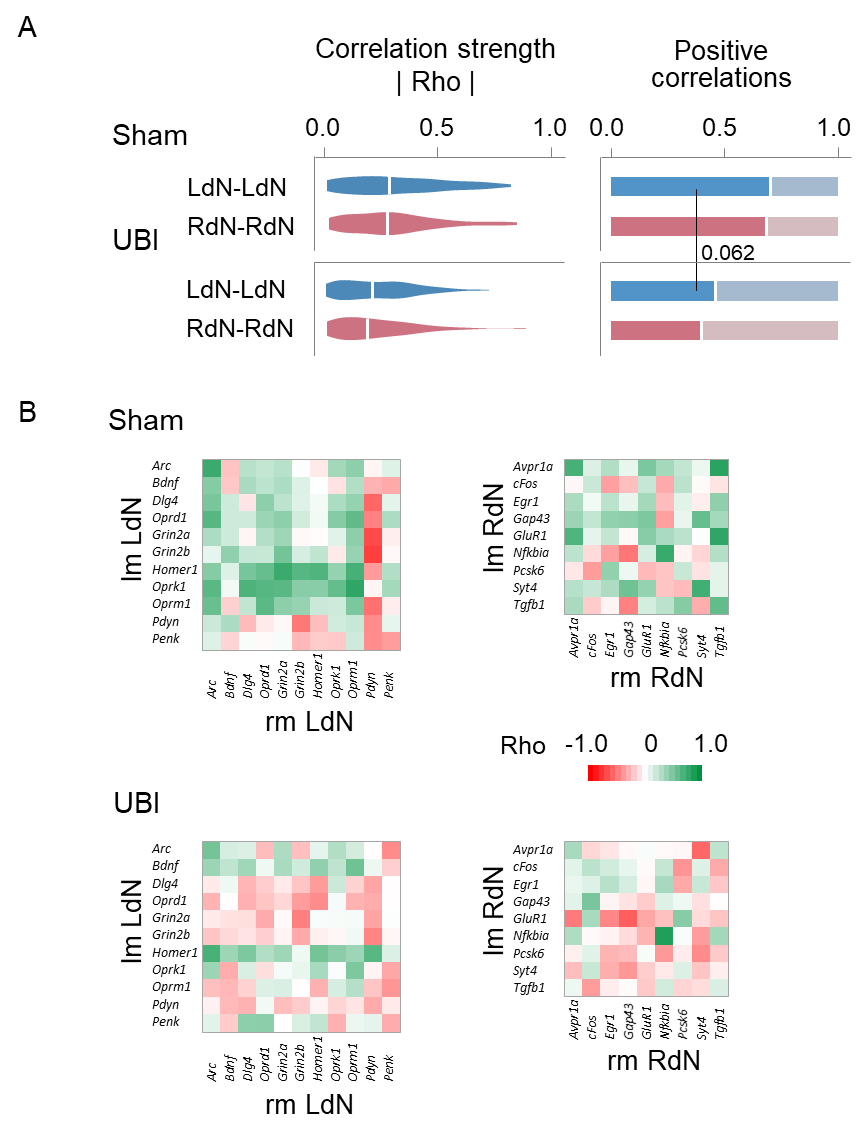


**Figure 5—figure supplement 2.** **Inter-modular correlations in the spinal cord of the sham surgery and UBI rats.** Inter-modular correlations (lm – rm) are visualized in **Figure 4—figure supplement 10**. (**A**) Comparison of the coordination strength and the proportion of positive correlations between the inter-modular internal LdN correlations and the inter-modular internal RdN correlations, and for both networks between the sham surgery and UBI groups. P values were determined by permutation testing with Benjamini-Hochberg family-wise multiple test correction. Significance for contrasts was determined by analysis of three AI_L/R_ categorization variants (**Figure 5—figure supplement 1**); P value is shown for the categorization variant with the median AI_L/R_ of the combined sham surgery and UBI group. (**B**) Heatmaps for Spearman’s rank coefficients for pairwise gene-gene the inter-modular correlations of the LdN and RdN.

**Source data:** The EXCEL source data file “SpinalC_SO_UBI_Ctrl_RD_DD.xlsx; Table III-S6 23 05 10.xlsx; raw_groups.xlsx”.


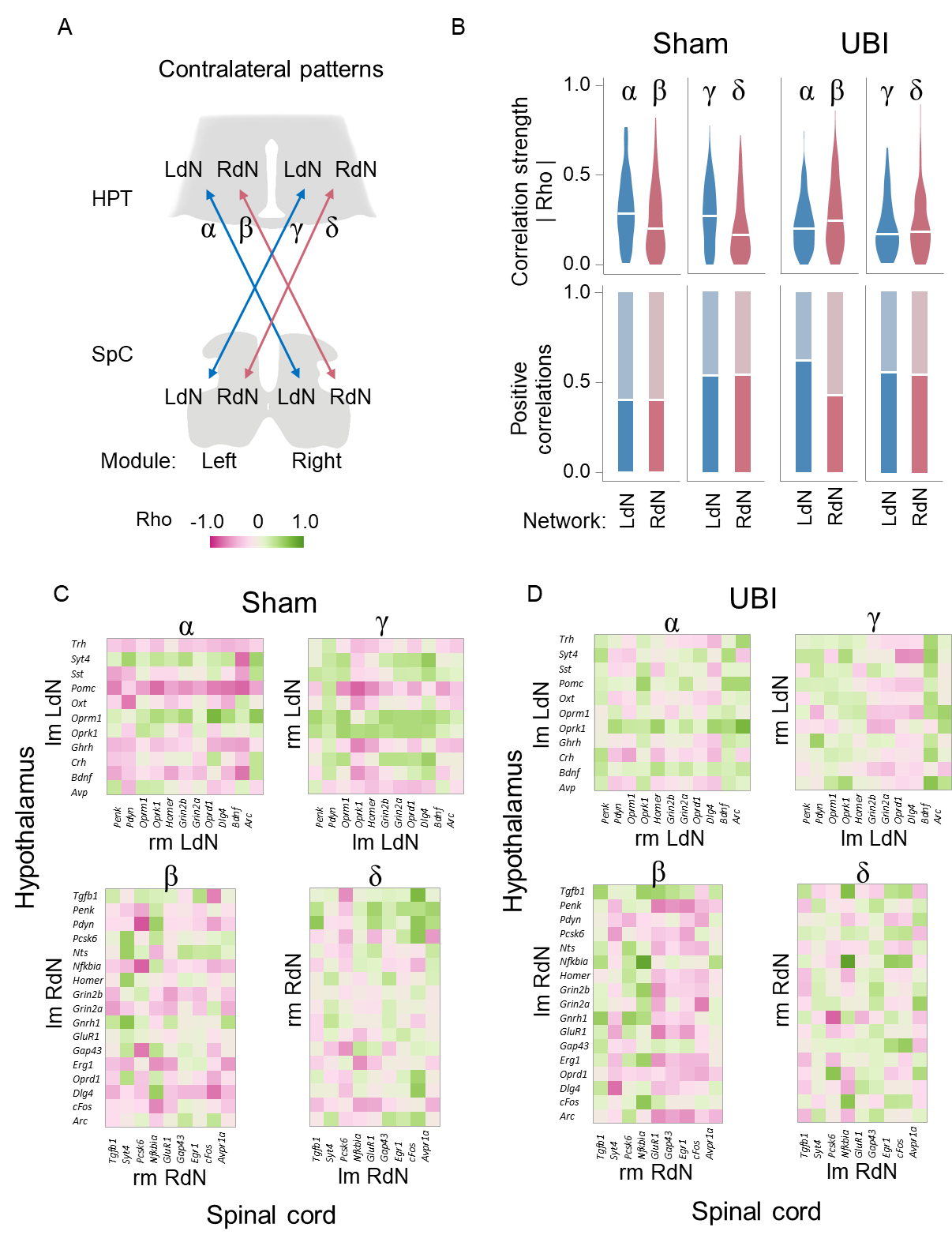


**Figure 6—figure supplement 1**. **Analysis of contralateral coordination of the LdN and RdN between the hypothalamus and lumbar spinal cord. The effects of UBI.** The experimental design and computation of LdN and RdN are described in **Figs. 4** and **5**. (**A**) Analyzed patterns of the contralateral pairwise gene-gene Spearman rank correlations between the left hypothalamus and right spinal cord (α and β), and between right hypothalamus and left spinal cord (γ and δ). (**B**) The coordination strength and the proportion of positive correlations for the correlation patterns depicted in **A**. The correlation patterns were compared between the LdN and RdN (α *vs*. β; γ *vs*. δ); each of them between the left and right modules (α *vs*. γ; β *vs*. δ), and all four patterns individually between UBI and sham surgery groups. P values were determined by permutation testing with Benjamini-Hochberg family-wise multiple test correction. Significance for contrasts was determined by analysis of three AI_L/R_ categorization variants (**Figure 4—figure supplement 9; Figure 5—figure supplement 1**). (**C**,**D**) Heatmaps for Spearman’s rank coefficients for pairwise gene-gene correlations for the left- (lm) and right (rm) modules in sham surgery and UBI groups.

**Source data:** The EXCEL source data file “Hypoth_SO_UBI.xlsx; SpinalC_SO_UBI_Ctrl_RD_DD.xlsx; Table III-S6 23 05 10.xlsx; raw_groups.xlsx”.
